# Modeling Mosquito-Borne Disease Spread in U.S. Urbanized Areas: The Case of Dengue in Miami

**DOI:** 10.1371/journal.pone.0161365

**Published:** 2016-08-17

**Authors:** Michael A. Robert, Rebecca C. Christofferson, Noah J. B. Silva, Chalmers Vasquez, Christopher N. Mores, Helen J. Wearing

**Affiliations:** 1 Department of Biology, University of New Mexico, Albuquerque, NM, United States of America; 2 Department of Mathematics and Statistics, University of New Mexico, Albuquerque, NM, United States of America; 3 Department of Pathobiological Sciences, Louisiana State University, Baton Rouge, LA, United States of America; 4 Miami-Dade County Mosquito Control Division, Miami, FL, United States of America; Centro de Pesquisas René Rachou, BRAZIL

## Abstract

Expansion of mosquito-borne pathogens into more temperate regions of the world necessitates tools such as mathematical models for understanding the factors that contribute to the introduction and emergence of a disease in populations naïve to the disease. Often, these models are not developed and analyzed until after a pathogen is detected in a population. In this study, we develop a spatially explicit stochastic model parameterized with publicly available U.S. Census data for studying the potential for disease spread in Urbanized Areas of the United States. To illustrate the utility of the model, we specifically study the potential for introductions of dengue to lead to autochthonous transmission and outbreaks in a population representative of the Miami Urbanized Area, where introductions of dengue have occurred frequently in recent years. We describe seasonal fluctuations in mosquito populations by fitting a population model to trap data provided by the Miami-Dade Mosquito Control Division. We show that the timing and location of introduced cases could play an important role in determining both the probability that local transmission occurs as well as the total number of cases throughout the entire region following introduction. We show that at low rates of clinical presentation, small outbreaks of dengue could go completely undetected during a season, which may confound mitigation efforts that rely upon detection. We discuss the sensitivity of the model to several critical parameter values that are currently poorly characterized and motivate the collection of additional data to strengthen the predictive power of this and similar models. Finally, we emphasize the utility of the general structure of this model in studying mosquito-borne diseases such as chikungunya and Zika virus in other regions.

## Introduction

Higher global connectivity and the increased volume of international travel have escalated the threat of importation, establishment, and expansion of many arboviral diseases [1–3]. The past two decades alone have been witness to the emergence of dengue, chikungunya, West Nile, Japanese encephalitis, and most recently, Zika viruses in naïve populations across the globe [1, 4–7]. When importation of these viruses leads to outbreaks, local healthcare systems can be overwhelmed, and resources for combating the spread of the virus are potentially inefficiently allocated. As the threat of arbovirus emergence increases, so does the need for adequate tools (such as mathematical models) for understanding the emergence potential and subsequent spread in naïve populations. These models help identify the key factors that lead to the spread of a virus and thereby inform policies aimed at mitigating the impacts on public health [8].

While these models are needed, there is a scarcity of data available for the model parameterization necessary for predicting outbreaks in populations in which a disease has never (or rarely) been present. Although this complicates the ability to make conclusive predictions regarding the impact of an introduced disease on a naïve population, models remain useful for developing a better understanding of which factors are critical for an introduction to lead to an outbreak [8]. A number of mechanistic models have been utilized recently to study factors that influence disease spread into new regions as well as to make predictions regarding the potential for introduced infectious diseases to become established in novel areas [9–14]. For example, following the introduction of chikungunya into the Western Hemisphere in late 2013, modeling studies were conducted to help understand the potential for spread throughout the Caribbean and the Americas [11, 12]. With no history of chikungunya in these regions, these studies focused on the impact that typical drivers of mosquito-borne disease spread, such as human travel and mosquito abundance, could have on the probability of chikungunya emergence. These studies were also aided by insights gained from a richer history of studying dengue, which is spread by the same vectors, throughout this region [6, 15].

Human movement via air and sea travel is often implicated as one of the primary factors in the global spread of mosquito-borne disease [3, 16–20], as well as the global spread of some disease vectors such as *Aedes aegypti* and *Aedes albopictus*, the mosquito vectors of dengue, chikungunya, yellow fever, and Zika viruses [21–26]. Human movement patterns also contribute to the spread of mosquito-borne disease at a finer scale, such as within cities [27–29]. Stoddard et al. [27] showed that movement of people among houses within Iquitos, Peru was a major driver of the spread of dengue throughout the city, and Adams and Kapan [29] demonstrated in a theoretical study that human movement across subpopulations may contribute to the persistence of mosquito-borne pathogens in urban areas. These studies emphasize the need to consider the role of human movement not only in the importation of mosquito-borne disease into naïve populations, but also in the spread of a pathogen throughout well-connected populations within urban areas. Unfortunately, data sets that describe human activity within and across subpopulations of large urban areas are rare, so approaches for approximating the movement of humans in infectious disease models have been borrowed from transportation theory. One common model to estimate the flow of people among populations is the gravity model [30]. The gravity model has been used in a number of previous studies to characterize human movement at the county level [31–34].

In this study, we develop a spatially explicit model that utilizes the gravity model to implicitly incorporate human movement into the probability of transmission to study the potential for mosquito-borne disease emergence in an Urbanized Area (UA) of the United States. As a case study, we parameterize the model to be representative of the Miami UA, and we describe mosquito dynamics in the model by fitting a population dynamic model to a unique data set provided by the Miami-Dade Mosquito Control Division. We use the model to explore the potential for importation of dengue to lead to emergence and spread within the Miami UA.

Dengue, which is vectored primarily by *Ae. aegypti*, was first reported in the Western Hemisphere in the 1800s and is now endemic to many countries in the Americas [1, 35]. Dengue is not thought to be currently established in any part of the United States; however, a number of large outbreaks occurred throughout the state of Florida in the first half of the 20th century, suggesting that the virus may have been established in the state during that period [35, 36]. Today, dengue is frequently imported into Florida from the many tropical and subtropical regions of the world to which it is endemic [35, 37]. From 2010 to 2015, there were 646 confirmed imported cases of dengue in Florida, 383 of which occurred within the Miami UA (S1 and S2 Figs) [37]. In 2009 and 2010, two outbreaks of locally transmitted dengue occurred in Key West, FL (Monroe County), with 28 locally acquired cases reported in 2009 and 56 in 2010 [37–39]. In 2013, another outbreak of 17 locally acquired cases was reported in Martin County, FL [37]. Smaller chains of local transmission have been reported in each of the past six years in parts of the Miami UA, but no outbreaks (which, for this study, we define to be more than 10 locally acquired cases that are related) were reported [37, 38].

Despite the few locally acquired cases of dengue reported in the Miami UA to date, it is likely one of the regions within the U.S. most at risk of a dengue outbreak. In addition to potentially being a dengue endemic region in the past, southern Florida is now home to both the primary and secondary (*Ae. albopictus*) vectors of the virus [36, 40–45]. The Miami UA also has a tropical climate with little fluctuation in temperature and precipitation throughout the year, which supports a suitable environment for both dengue virus and its vectors and is similar to the climate of many dengue endemic regions of the world. Furthermore, the Miami UA is highly connected to dengue endemic countries in the Caribbean and Latin America via three international airports and four passenger and cargo seaports. In 2013, Miami International Airport and Fort Lauderdale-Hollywood International Airport were the 16^th^ and 22^nd^ busiest U.S. airports, respectively, by passengers enplaned [46]. Also in 2013, the Miami International Airport was listed as the second busiest U.S. airport for international travel and the top gateway to Latin America and the Caribbean [47]. The Port of Miami and Port Everglades in Fort Lauderdale are the two busiest seaports in the U.S. for cruise travel [46], and the majority of destinations for ships departing these ports are in the Caribbean and Central America [48].

To demonstrate the utility of this model for detecting factors that could lead to dengue emergence in the Miami UA, we study the impact of mosquito population dynamics and human movement on the potential for emergence by investigating the influence of timing and location of introduction on both the probability of autochthonous transmission occurring and the size of outbreaks following successful local transmission. We further explore the influence of clinical presentation rates on the ability to detect local transmission and outbreaks, and we discuss sensitivity of the model to parameters that are poorly characterized for the Miami UA.

## Materials and Methods

### Model Description

We employ a vector-host epidemic model with discrete time and discrete state space. The dynamics of the system are described by an adaptation of a Reed-Frost chain binomial system to incorporate demographic stochasticity [49]. As the frequency at which local cases of dengue in the Miami UA are reported is low (S2 Fig), we have developed this model to capture the impacts of stochastic effects on the probability of emergence and outbreaks of dengue.

We track the dynamics of one serotype of dengue in humans and adult female *Ae. aegypti* populations in the 186 cities, census designated places (CDPs), and census county divisions (CCDs) that comprise the Miami UA. Note that in this paper we are interested in studying the potential for an outbreak following an introduction of a single serotype, so we ignore the potential for introduction and circulation of multiple serotypes. Throughout, we denote classes of the human population with the subscript *H* and *Ae. aegypti* classes with the subscript *G*. At each location *i* on each day *t*, we divide the human population into susceptible (*S*_*H*_(*i*, *t*)), exposed (*E*_*H*_(*i*, *t*)), infectious (*I*_*H*_(*i*, *t*)), and recovered (*R*_*H*_(*i*, *t*)) classes. The total human population in location *i* at time *t* is denoted by *N*_*H*_(*i*, *t*) = *S*_*H*_(*i*, *t*) + *E*_*H*_(*i*, *t*) + *I*_*H*_(*i*, *t*) + *R*_*H*_(*i*, *t*). Throughout the study, we are primarily interested in the potential for emergence and outbreaks within a year of introduction of the virus, so we neglect human births and deaths as well as immigration and emigration in the region and assume that the human population in each location remains constant throughout the year so that *N*_*H*_(*i*, *t*) = *N*_*H*_(*i*) for all *t*. Although rates of travel into and out of the region may vary seasonally, we do not anticipate this having a significant impact on the human population size.

Similar to the compartmentalization of the human population, we divide each vector population into susceptible (*S*_*G*_(*i*, *t*)), exposed (*E*_*G*_(*i*, *t*)), and infectious (*I*_*G*_(*i*, *t*)) classes. The total vector population in each location *i* at time *t* is denoted by *N*_*G*_(*i*, *t*) = *S*_*G*_(*i*, *t*) + *E*_*G*_(*i*, *t*) + *I*_*G*_(*i*, *t*).

Stochastic dynamics for humans are governed by the probabilities of becoming infected, becoming infectious, and recovering from infection. We define all probabilities on a daily time interval (i.e. on the time interval [*t*, *t* + 1]); however, time intervals of different lengths could be considered by rescaling parameter values. We define the vector-to-host transmission rate (biting rate × probability of a bite leading to transmission of the virus) as *β*. In a single location *i* in the absence of human movement in the region, the probability that a susceptible human becomes infected is given by

$$\lambda_{H}\left(i,t\right)=1-\exp\left(-\frac{\beta I_{G}\left(i,t\right)}{N_{H}\left(i\right)}\right)$$ (1)

Once infected, the probability that a host becomes infectious is given by *σ*_*H*_, which is defined in terms of the average intrinsic incubation period, ${\left({\hat{\sigma }}_{H}\right)}^{-1}$:

$$\sigma_{H}=1-\exp\left(-{\hat{\sigma }}_{H}\right)$$ (2)

The probability that an infectious host recovers is given by *γ*_*H*_, which is defined in terms of the average duration of infectiousness for humans, ${\left({\hat{\gamma }}_{H}\right)}^{-1}$:

$$\gamma_{H}=1-\exp\left(-{\hat{\gamma }}_{H}\right)$$ (3)

Taken together, the human dynamics in the absence of movement about the region are given by the following equations:

$$W_{H}\left(i,t\right)∼\text{binomial}\left(S_{H}\left(i,t\right),\lambda_{H}\left(i,t\right)\right)$$ (4)

$$V_{H}\left(i,t\right)∼\text{binomial}\left(E_{H}\left(i,t\right),\sigma_{H}\right)$$ (5)

$$U_{H}\left(i,t\right)∼\text{binomial}\left(I_{H}\left(i,t\right),\gamma_{H}\right)$$ (6)

$$S_{H}\left(i,t+1\right)=S_{H}\left(i,t\right)-W_{H}\left(i,t\right)$$ (7)

$$E_{H}\left(i,t+1\right)=E_{H}\left(i,t\right)+W_{H}\left(i,t\right)-V_{H}\left(i,t\right)$$ (8)

$$I_{H}\left(i,t+1\right)=I_{H}\left(i,t\right)+V_{H}\left(i,t\right)-U_{H}\left(i,t\right)$$ (9)

$$R_{H}\left(i,t+1\right)=R_{H}\left(i,t\right)+U_{H}\left(i,t\right).$$ (10)

Here, *W*_*H*_(*i*, *t*), *V*_*H*_(*i*, *t*), and *U*_*H*_(*i*, *t*) represent the number of newly infected, newly infectious, and newly recovered individuals, respectively, in location *i* at time *t*.

Unlike the human population, the vector population varies daily throughout the year due to a seasonally varying per capita recruitment rate, *ψ*_*G*_(*t*), where

$$\psi_{G}\left(t\right)=\mu_{G}\left(1+\nu \cos\left(\frac{2\pi }{365}\left(t-\tau_{G}\right)\right)\right)$$ (11)

In this equation, *ν* and *τ*_*G*_ determine the amplitude and the timing, respectively, of the peak recruitment rate during the season. The parameter *μ*_*G*_ is the probability of vector mortality each day. In order to maintain a stable average population size, we also set the average per capita recruitment rate to be *μ*_*G*_. The recruitment rate is defined as the average number of adult female mosquitoes produced by a single adult female mosquito each day (the number of eggs laid, survival through juvenile stages, and sexing of offspring is modeled implicitly in this recruitment rate). We define *μ*_*G*_ as

$$\mu_{G}=1-\exp\left(-{\hat{\mu }}_{G}\right)$$ (12)

where ${\left({\hat{\mu }}_{G}\right)}^{-1}$ is the average lifespan of *Ae. aegypti* in days.

Stochastic dynamics for vectors are governed by the probabilities of recruitment, death, becoming infectious, and becoming infected. The number of new vectors recruited in location *i* at time *t* is chosen from a Poisson distribution with mean *ψ*_*G*_(*t*)*N*_*G*_(*i*, *t*). That is, if *M*_*G*_(*i*, *t*) is the number of newly recruited adult vectors in location *i* at time *t*, then

$$M_{G}\left(i,t\right)∼\text{Poisson}\left(\psi_{G}\left(t\right)N_{G}\left(i,t\right)\right)$$ (13)

In the absence of sufficient data to suggest differences in human-to-vector and vector-to-human transmission rates, we define the human-to-vector transmission rate to be equal to the vector-to-human transmission rate, *β*. The probability that a susceptible vector becomes infected in location *i* at time *t* is

$$\lambda_{G}\left(i,t\right)=1-\exp\left(-\frac{\beta I_{H}\left(i,t\right)}{N_{H}\left(i\right)}\right)$$ (14)

A susceptible vector can either remain susceptible, become infected and live to the next day, or die (either infected or uninfected). We assume that death is independent of becoming infected or becoming infectious. The probability that a susceptible vector becomes infected and lives to the next day is (1 − *μ*_*G*_)*λ*_*G*_(*i*, *t*). The number of susceptible vectors that either progress to the infected class, die, or remain susceptible is chosen from a multinomial distribution. Let *W*_*G*_(*i*, *t*) be the number of vectors in location *i* at time *t* that become infected and survive and *X*_*S*_(*i*, *t*) be the number of susceptible vectors in location *i* at time *t* that die. Then,

$$\left[W_{G}\left(i,t\right),X_{S}\left(i,t\right)\right]∼\text{multinomial}\left(S_{G}\left(i,t\right),\left[\left(1-\mu_{G}\right)\lambda_{G}\left(i,t\right),\mu_{G}\right]\right)$$ (15)

Once infected, the probability that a vector becomes infectious is *σ*_*G*_, which is defined in terms of the average extrinsic incubation period, ${\left({\hat{\sigma }}_{G}\right)}^{-1}$:

$$\sigma_{G}=1-\exp\left(-{\hat{\sigma }}_{G}\right)$$ (16)

The probability that a vector becomes infectious and lives to the next day is (1 − *μ*_*G*_)*σ*_*G*_. The number of infected vectors that either progress to the infectious class, die, or remain infected is chosen from a multinomial distribution. Let *U*_*G*_(*i*, *t*) be the number of vectors that become infectious in location *i* at time *t* and *X*_*E*_(*i*, *t*) be the number of exposed vectors in location *i* at time *t* that die. Then,

$$\left[U_{G}\left(i,t\right),X_{E}\left(i,t\right)\right]∼\text{multinomial}\left(E_{G}\left(i,t\right),\left[\left(1-\mu_{G}\right)\sigma_{G},\mu_{G}\right]\right).$$ (17)

Once infectious, a vector dies with probability *μ*_*G*_. The number of infectious vectors that die is chosen from a binomial distribution. Let *X*_*I*_(*i*, *t*) be the number of infectious vectors in location *i* at time *t* that die. Then,

$$X_{I}\left(i,t\right)∼\text{binomial}\left(I_{G}\left(i,t\right),\mu_{G}\right).$$ (18)

The mosquito dynamics in the absence of human movement are given by the following equations:

$$S_{G}\left(i,t+1\right)=S_{G}\left(i,t\right)+M_{G}\left(i,t\right)-W_{G}\left(i,t\right)-X_{S}\left(i,t\right)$$ (19)

$$E_{G}\left(i,t+1\right)=E_{G}\left(i,t\right)+W_{G}\left(i,t\right)-U_{G}\left(i,t\right)-X_{E}\left(i,t\right)$$ (20)

$$I_{G}\left(i,t+1\right)=I_{G}\left(i,t\right)+U_{G}\left(i,t\right)-X_{I}\left(i,t\right)$$ (21)

### Type Reproductive Number

For this model, we define the time-varying type reproductive number [50] in a single population *i* to be $R_{0}^{T}\left(i,t\right)$, where

$$R_{0}^{T}\left(i,t\right)=\frac{\beta^{2}\sigma_{G}\left(1-\mu_{G}\right)N_{G}\left(i,t\right)}{\left(\sigma_{G}\left(1-\mu_{G}\right)+\mu_{G}\right)\mu_{G}\gamma_{H}N_{H}\left(i\right)}$$ (22)

Refer to S1 Text for details of this calculation. In S3 Fig, we show how this value varies across the year with fluctuations in the vector population. We note that once movement is incorporated into the probability of infection term, the global value of $R_{0}^{T}\left(t\right)$ will be different from the value given in Eq 22, but this value provides a good approximation.

### Implicit Human Movement

We model movement of humans about the Miami UA implicitly by including a term within the probabilities of infection *λ*_*H*_(*i*, *t*) and *λ*_*G*_(*i*, *t*) that allows for infection to be acquired via contact in locations throughout the region. The probability that an infection is acquired from another location depends upon the rate of movement between locations, which is approximated by the gravity model [30]. The general premise of the gravity model is that movement between locations is proportional to the population size of the two locations and inversely proportional to the distance between locations. There are three parameters that determine the degree of this relationship. Namely, *a* and *b*, which determine the degree of attractiveness of the source and destination populations, respectively, and *c*, which determines the impact of the distance between the two populations. For population *i* of size *N*_*i*_ and population *j* of size *N*_*j*_, the daily flux from *i* to *j* is given by *m*_*ij*_, where

$$m_{ij}=\frac{{\left(N_{i}\right)}^{a}{\left(N_{j}\right)}^{b}}{{\left(d_{ij}\right)}^{c}}$$ (23)

Here, *d*_*ij*_ is the distance between populations *i* and *j*. For this study, we calculate the distance between two populations using the Haversine formula (refer to S1 Text for details). We estimated the values of parameters *a*, *b*, and *c* by fitting the gravity model to daily commute data obtained from the U.S. Census [51]. Details of the parameter estimation can be found in S1 Text.

With implicit human movement incorporated, the probability that a human in location *i* at time *t* becomes infected is now

$$\lambda_{H}\left(i,t\right)=1-\exp\left(-\frac{\beta I_{G}\left(i,t\right)}{N_{H}\left(i\right)}-\theta {\left(N_{H}\left(i\right)\right)}^{a}\sum_{j\neq i}\frac{{\left(N_{H}\left(j\right)\right)}^{b}}{{\left(d\left(j,i\right)\right)}^{c}}\cdot \frac{\beta I_{G}\left(j,t\right)}{N_{H}\left(j\right)}\right),$$ (24)

and the probability that a vector in location *i* at time *t* becomes infected is now

$$\lambda_{G}\left(i,t\right)=1-\exp\left(-\frac{\beta I_{H}\left(i,t\right)}{N_{H}\left(i\right)}-\theta {\left(N_{H}\left(i\right)\right)}^{a}\sum_{j\neq i}\frac{{\left(N_{H}\left(j\right)\right)}^{b}}{{\left(d\left(j,i\right)\right)}^{c}}\cdot \frac{\beta I_{H}\left(j,t\right)}{N_{H}\left(j\right)}\right).$$ (25)

In these two terms, the parameter *θ* does not have a biological or demographic interpretation, but determines the proportional impact of movement on the probability of acquiring infection. The value of *θ* is on the order of magnitude of the inverse of the total population size in the region; this parameter then summarizes the overall impact of human movement on new infections throughout the entire region. This characterization of the impact of human movement on acquiring infections assumes that a susceptible human in population *i* can become infected by contact with an infectious vector in population *i* or contact with an infectious vector in another population *j*. Similarly, a susceptible vector in population *i* can become infected by an infectious human in population *i* or an infectious human that is visiting from another population *j*. We assume here that the impact of movement is independent of whether a human is susceptible or infectious, thus the value of *θ* is the same in both terms. Note here that we assume that the movement about various locations does not significantly affect the population sizes in each location.

We remark that for the purposes of this study, we do not consider the impacts of movement of the vector population. Mark-release-recapture studies have found that *Ae. aegypti* females do not disperse great distances, typically staying within 50–100 meters of the release site [52–54]. Because the spatial structure of the population in our model is coarse (that is, at the level of Census Designated Places (CDPs) and not houses within neighborhoods), we do not anticipate mosquito movement contributing to the spread of dengue from one location to another.

### Parameterization of human population structure and movement

As of the 2010 U.S. Census, the Miami UA consists of the majority of Miami-Dade, Palm Beach, and Broward counties as well as part of southern Martin County [55]. Within the four counties, there are 186 cities, towns, villages, CDPs, and unincorporated regions of census county divisions (CCDs) that are considered to be a part of the Miami UA (see S4 Fig) [55]. The Miami UA has a total population size of 5.5 million people and a population density of 1715.72 people per square km of land [55]. The human population size of the 186 locations within the region ranges from 1 (the unincorporated area of Pompano Beach CCD) to 399,443 (Miami) with a median size of 13,517. S4 Fig illustrates the 186 regions and the heterogeneity in population size across the area. In our model, we include populations in the 161 different CDPs and 25 unincorporated populations within CCDs. We obtained human population size data from U.S. Census Bureau population and housing tables from the 2010 census [55]. Human daily commute data was obtained from the U.S. Census Bureau OnTheMap tool [51]. For a detailed description of U.S. Census data acquisition, see S1 Text. To estimate parameter values in the gravity model, we fit Eq 23 to the daily commute data utilizing a nonlinear least squares approach. We remark that utilizing the gravity model rather than the raw daily commute data was necessary because the U.S. Census Bureau OnTheMap tool includes only the 161 CDPs and not the 25 unincorporated populations within the CCDs. Further details of the gravity model fitting are included in S1 Text and error associated with fitting is shown in S5 Fig.

### Parameterization of mosquito distribution and seasonality

The Miami-Dade County Mosquito Control Division placed CDC light traps in various locations throughout Miami-Dade county during 2010–2013 (S6 Fig). Of all of the traps, only ten traps were present at the same locations across the four years, and we chose to analyze data for these ten traps to estimate the average seasonal behavior of the *Ae. aegypti* populations during this time period. We fit a deterministic model of mosquito population dynamics to data from each of the traps to characterize the seasonality of the vector population. We utilized a generalized least squares approach to estimate a value for *τ*_*G*_, which determines the time at which the mosquito population reaches its peak each year (see Eq 11). Of the ten traps, data from two of the traps were too sparse to capture seasonal variation (see S1 Text for details), so we estimated the mean value for *τ*_*G*_ from eight different model fits. The mean value of *τ*_*G*_ was 90.89 days with a 95% confidence interval of (81.01, 100.77). S1 Text contains a detailed description of the process of estimating *τ*_*G*_. Additionally, S7 Fig shows the dynamics of the population model fit to the trap data for each of the traps. We note that the timing of seasonal peaks and troughs observed with this estimate of *τ*_*G*_ (S8 Fig) are in agreement with those observed in a recent previous study in Palm Beach county [45].

Throughout this paper, we assume a vector-host ratio that varies seasonally according to Eq 11, but is constant across the subpopulations. S8 Fig shows the deterministic population dynamics of vectors for different values of average vector-to-host ratios. We note that in this study the average vector-host ratio is defined as the ratio of vectors to humans averaged across a single year, and this value is determined by *a priori* simulations of population dynamics in a deterministic model. We implemented a generalized least squares approach to estimate the value of the initial population size of *Ae. aegypti* (and thus the initial vector-host ratio) that would generate the desired average vector-host ratio in a homogeneously mixed human population when we simulated one year of vector population dynamics with the deterministic model given in Eq 26. We then initialized the vector populations in each of the 186 locations within the Miami UA by calculating the initial population size of the vectors as the product of the initial vector host ratio and the human population size at each location.

$$N_{G}\left(t+1\right)=N_{G}\left(t\right)+\mu_{G}N_{G}\left(t\right)\left(1+\nu \cos\left(\frac{2\pi }{365}\left(t-\tau_{G}\right)\right)\right)-\mu_{G}N_{G}\left(t\right)$$ (26)

### Parameter Selection

We utilized the data in the Florida Department of Health reports (S1 and S2 Figs, [37]) to assist in selecting some parameter values for our model. Fixing all other parameters at the values listed in Table 1, we first conducted a global sensitivity analysis of three poorly characterized parameters: the average vector-host ratio; the transmission rate, *β*; and the gravity model proportionality coefficient, *θ*. We generated 5000 sets of values for these parameters, each value randomly and independently chosen from three distinct uniform distributions (the average vector-host ratio ∼ Uniform(0, 3), *β* ∼ Uniform(0, 4), and log_10_(*θ*) ∼ Uniform(−8, −4)). For each parameter set, we conducted 100 simulations wherein one infectious person was introduced in the city of Miami on May 30. We calculated the probability of autochthonous transmission following introduction as well as the median number of cases that occurred within 100 days of introduction when autochthonous transmission did occur. (We chose 100 days to maintain a standard epidemiologically relevant measure of time following introduction throughout the entirety of the study). We then grouped parameter values and compared the cumulative distribution of each of these two metrics from simulations across parameter groups. Detailed results of the sensitivity analysis are presented below in the last section of the Results.

**Table 1:** Table of default parameter values.

Parameter	Description	Default Value	Source
*β*	Vector to human transmission rate	0.16	Est.
${\left({\hat{\sigma }}_{G}\right)}^{-1}$	Average extrinsic incubation period	9	[56]
${\left({\hat{\mu }}_{G}\right)}^{-1}$	Average vector lifespan	10	[52, 57]
*ν*	Amplitude of the vector recruitment seasonality function	0.25	Est.
*τ*_*G*_	Phase shift of the vector recruitment seasonality function	90.89	SM
	Average vector-host ratio in each location	1	Est.
*β*	Human to vector transmission rate	0.16	Est.
${\left({\hat{\sigma }}_{H}\right)}^{-1}$	Average intrinsic incubation period	4	[58]
${\left({\hat{\gamma }}_{H}\right)}^{-1}$	Average duration of infectiousness	7	[59, 60]
*θ*	Gravity model proportionality coefficient	3 × 10^−6^	Est.
*a*	Gravity model donating population exponent	0.296	SM
*b*	Gravity model receiving population exponent	0.4371	SM
*c*	Gravity model distance exponent	0.749	SM
	Default location of dengue introduction	Miami, Florida	
	Default day of dengue introduction	150 (May 30)	
	Default number of infectious individuals introduced	1	

Parameters marked ‘Est.’ are estimated from the parameter selection process described in the text. For parameters marked SM, refer to S1 Text for details regarding the estimation of the values shown here.

To select values for these three parameters, we determined plausible parameter sets utilizing an approach similar to that described in [61]. For each of the 5000 parameter sets, we compared the model output for the total number of cases that occurred within 100 days of introduction to the number of cases that occurred in the 2013 Martin County outbreak (S1 and S2 Figs). We assumed that only 5–10% of cases in the Martin County outbreak were reported (based on calculations from data presented in [39] and [62] following the 2009-2010 Key West Outbreak), and selected sets of values of the average vector-host ratio, *θ*, and *β* whose maximum outbreak size was within a range of 170–340 total cases when an introduction occurred near the peak of the season (May 30). Here we chose to compare the maximum value of the simulations because we assume that, due to low reporting rates, the outbreaks that have occurred represent the extremes of local transmission events. The interval 170–340 was calculated by assuming that only 5–10% of the 17 cases that occurred in Martin county were reported. We note that we chose to utilize Martin County for the selection of parameters because the region in which the 2012 outbreak occurred is more representative of parts of the Miami UA than is Key West, the only other location in Southern Florida where an outbreak has occurred in recent years (in fact, part of Martin County is included in the Miami UA).

We found that of the 5000 parameter sets, 154 (3.04%) resulted in a maximum number of cases in the interval of 170–340. We selected one parameter set of the 154 as the default values for this study (See Table 1). We note that because the data available are sparse and because we have a poor understanding of mosquito abundance in this region, multiple combinations of these three parameters can lead to similar outcomes. In S1 Text, we further explore the non-identifiability of these parameters. In particular, we show that in plausible sets of *β* and the average vector host ratio, values of the two parameters are proportional to one another (S9 Fig). Also in S1 Text, we show the distribution of the parameter values in the plausible parameter sets (S10 Fig).

### Scenarios and Metrics of Interest

Previous work has shown that the potential for disease outbreaks can be influenced by factors such as human population size, vector abundance, seasonality in transmission, and connectivity of populations [63–70]. In this study, we consider a number of scenarios to address the impact of some of these factors on outbreaks of dengue in the Miami UA. Each scenario that we consider has a unique parameter set. For example, a single scenario may have the default parameters listed in Table 1, except that the imported case occurs on July 11 rather than May 30. For each scenario, we conducted 500 simulations. We note that, for the purpose of this study, we are interested in the dynamics that follow a single introduction of dengue into the region, and we do not consider the impacts of multiple introductions. By only considering a single introduction, we can better understand the impacts of the factors that lead to successful local transmission and outbreaks. Furthermore, given the relatively low numbers of imported and locally acquired cases of dengue in the region, we assume an entirely susceptible population prior to the first introduction.

Throughout the results, we primarily present two metrics of epidemiological relevance: the probability of autochthonous transmission (i.e., the probability that an imported case leads to at least one locally acquired human case) and the total number of human cases that occur throughout the entire Miami UA within 100 days of the initial import. These two metrics are particularly important for preparing the public health community to respond to outbreaks and helping to inform policies for implementing vector control. We calculate the probability of autochthonous transmission by summing the number of simulations that lead to 1, 10, 50, 100, or 500 locally acquired cases within one year of an imported case and dividing by the total number of simulations conducted. These outbreak sizes were chosen to understand the potential for outbreaks of different sizes across different orders of magnitude. The total number of cases that occur within 100 days of the initial import are presented only for simulations in which at least one autochthonous case occurs.

## Results

### Timing of Introduction

**Fig 1:**
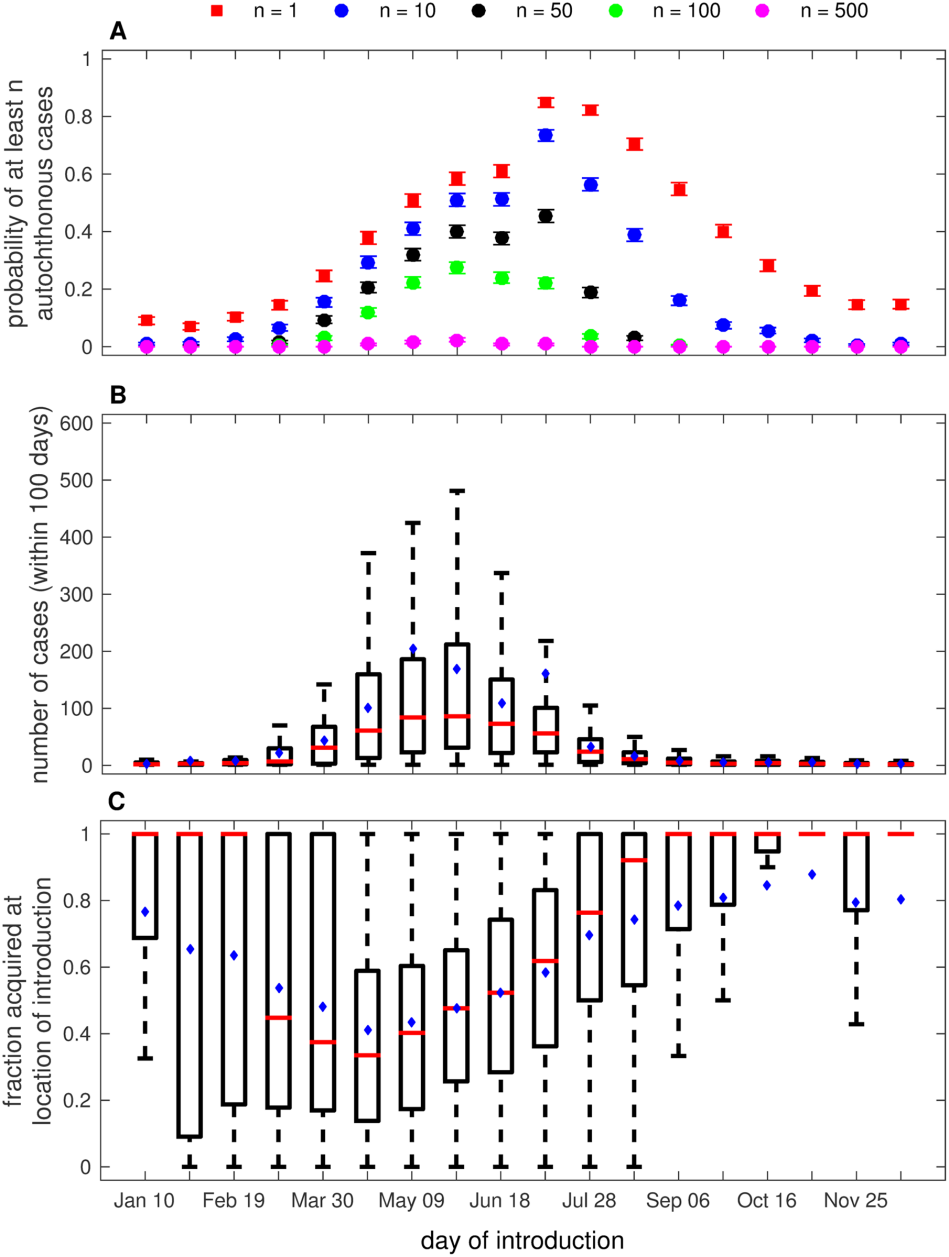
The impact of the timing of introduction on local transmission and outbreak. (A) The probability that at least *n* autochthonous cases occurred following a single introduction on the date given on the horizontal axis (error bars are the probability of autochthonous transmission ± the binomial standard deviation). (B) The total number of dengue cases that occurred within 100 days of a single introduction on the date listed along the horizontal axis. (C) The fraction of total cases that occurred in the location of introduction. All other parameter values are as given in Table 1. In the box plots in (B) and (C), red lines indicate the median, blue dots indicate the mean, and the box represents the interquartile range. Whiskers indicate the interquartile range multiplied by 1.5.

Because the *Ae. aegypti* population varies seasonally, we investigate the impacts of the time of year at which an imported case arrives in the region on the probability that local transmission occurs and the number of cases that follow a single introduction (Fig 1). We held all parameters at the values listed in Table 1 except for the day of introduction, and we introduced a single infectious person into the city of Miami on different days throughout the year, starting with January 10 and incrementing every 10 days until December 26. We calculated the probability that autochthonous transmission occurred (Fig 1A) and recorded the total number of cases that occurred in the 100 days following introduction (Fig 1B).

The probability of autochthonous transmission occurring increased from January until July and decreased from July until December. Imports that occurred between January and February led to at least one case caused by local transmission in fewer than 20% of simulations as did imports that occurred in late October and November and December. The probability that local transmission occurred increased through the spring months, and imports occurring between early June and early August led to local transmission in more than 60% of simulations, with the highest probability of local transmission following imports in late June (80%). The probability of local transmission remained above 50% following imports throughout August and early September, but decreased in the autumn months.

The probability of larger numbers of locally acquired cases occurring exhibited variation similar to that of the probability that at least one case occurred; however, the rate of decline in the probability that occurred following the peaks in May-July was greater for the probability of having a larger number of cases (e.g. compare *n* = 100 with *n* = 1 in Fig 1). The peak timing at which an introduction led to local cases also shifted to earlier in the year for the probability of larger numbers of cases. For example, the probability that at least 500 cases occurred was highest following imports in early May whereas the probability that at least 100 cases occurred was highest following imports in late May and early June.

The total number of cases that occurred within 100 days of an imported case varied across the year in much the same way that the probability of observing outbreaks of at least 500 cases did (Fig 1B). The median number of cases that occurred following imports from January to mid-March as well as mid-September to December was fewer than 10. The variation in these months was also low. The median number of cases and the variability in the number of cases that occurred increased for imports during April and May and decreased for imports that occurred in June and the following months. Imports in May led to the highest median number of cases (around 100), and the median number of cases was greater than 50 for imports that occurred in late April and June.

The differences in the probability that an autochthonous case occurred and the number of cases within 100 days following an imported case were driven by the *Ae. aegypti* population dynamics (see S8 Fig) which led to variation in the time-varying type reproductive number, $R_{0}^{T}\left(t\right)$ (S3 Fig), due to the direct relationship between $R_{0}^{T}\left(t\right)$ and the vector population size (see Eq 22). The probability that at least one locally acquired case occurred following an imported case was highest when the vector population was highest, and the probability increased and decreased with the vector population. Both the probability that larger numbers of locally acquired cases occurred and the total number of cases that occurred within 100 days of the initial introduction were highest when introductions occurred as the vector population was increasing towards its peak size in July. However, both of these metrics began decreasing when the imported cases arrived before the vector population reached its peak.

To assess the impact of movement throughout the region on outbreaks and how that impact varies with the timing of imported cases, we also calculated the fraction of total cases throughout the region that were acquired in the location of introduction (for these results, the city of Miami, Fig 1C). We found that when autochthonous transmission occurred, but the total number of cases was low, the majority of total cases were acquired in the location of introduction. As the outbreaks grew, the fraction of cases acquired at the location of introduction (FCALOI) decreased. In the larger outbreaks that occurred following introduction in late April and May, the median FCALOI was about 0.40, and the variability in the FCALOI was lower for introductions during this time than for introductions earlier in the year. In contrast, the small chains of transmission (typically fewer than 10 cases) that occurred following introductions in January-February and October-December had a median FCALOI of about 1. There was very little variability in FCALOI in October-December, but high variability in FCALOI in January and February. Although the median FCALOI was over 0.75 when introductions occurred in late July and August, the variability in FCALOI was high. This high variability was driven in part by the low, but variable, number of cases that occurred following imports during these months.

### Location of Introduction

**Fig 2:**
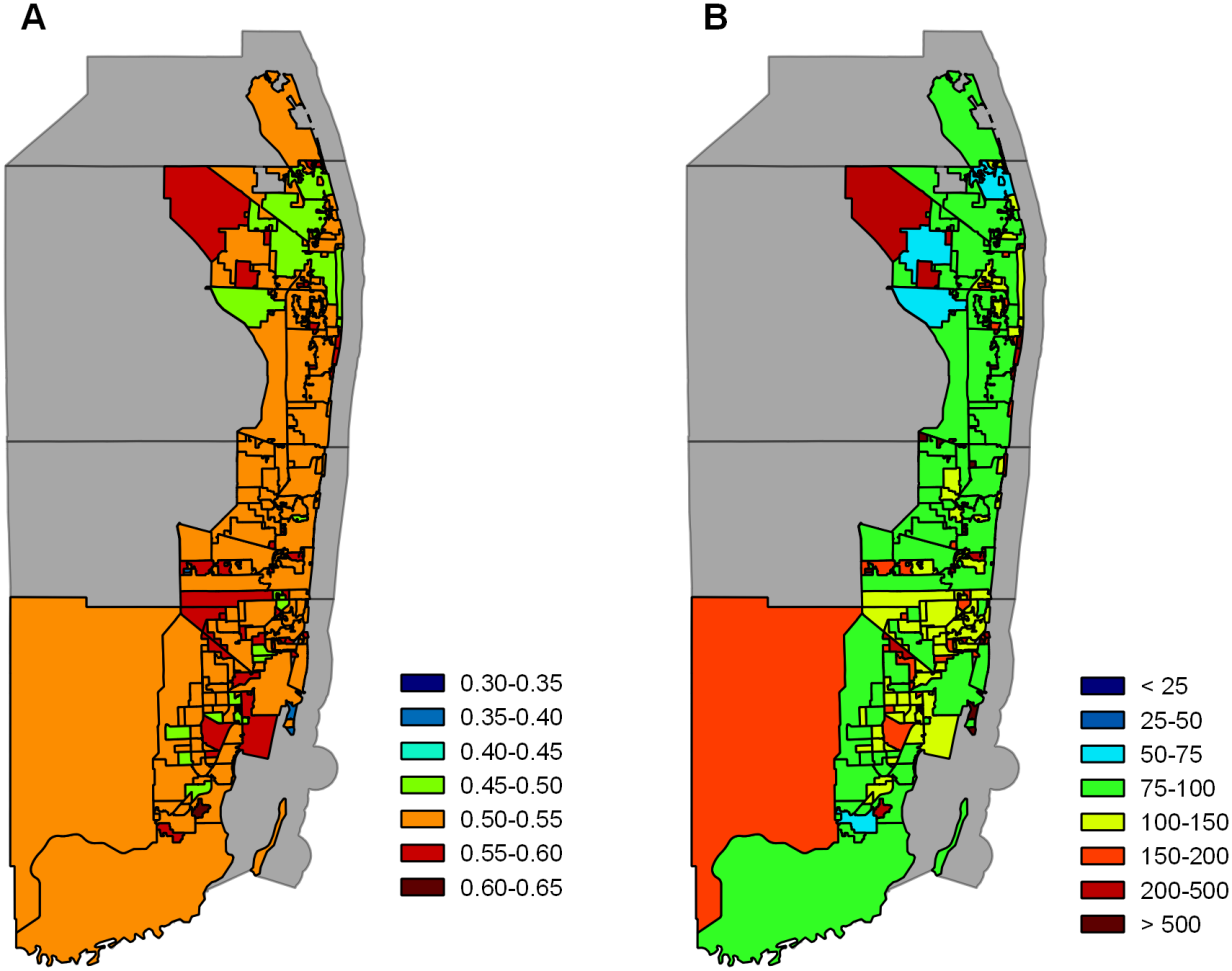
The impact of the location of introduction on local transmission and outbreak throughout the entire Miami UA. Probability that at least one autochthonous case occurred in the Miami UA (A) and the total number of dengue cases that occurred throughout the entire Miami UA in the first 100 days (B) following a single introduction on May 30 in each of the 186 locations. The metric presented on each map represents the metric for an introduction in that location. All other parameter values are as given in Table 1. The map included in this figure was obtained from U.S. Census Bureau TIGER/LINE^®^Shapefiles [71].

**Fig 3:**
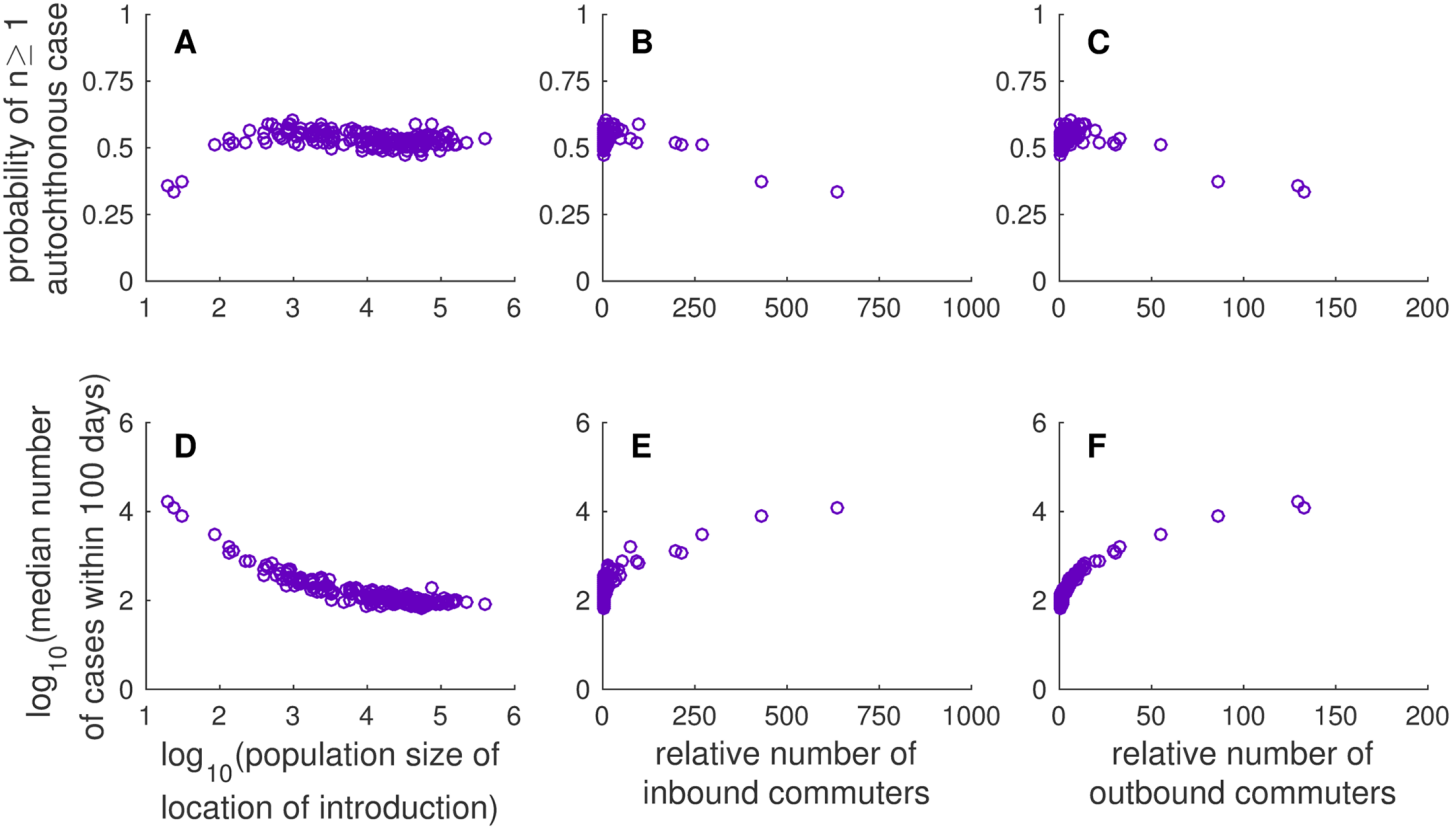
Scatter plots of relationships between location of introduction characteristics and metrics of outbreak probability and size. Each column shows a different population of introduction characteristic: (A,D) population size (log scale), (B,E) the number of commuters traveling to the population of introduction relative to the population size of the location, and (C,F) the number of commuters traveling from the location of introduction relative to the population size of the location. The first row (A-C) shows the probability of at least one autochthonous case, and the second row (D-F) shows the median number of cases that occurred within 100 days of introduction (log scale). These relationships were generated from the model output presented in the Fig 2.

Next, we address the impact that heterogeneity in human population size and movement have on the probability of autochthonous transmission and the total number of cases that occurred within 100 days of introduction (Fig 2). For this part of the study, we introduced a single infectious person into one of the locations within the Miami UA on May 30 and observed the number of cases that occurred throughout the entire region following this introduction. We then repeated this for each of the 186 different locations. To identify potential drivers of heterogeneity in results, we tested for correlations between our two primary metrics (the probability of at least one autochthonous case and the median number of cases that occurred within 100 days) and demographic characteristics of the CDPs where the initial import occurred (human population size, the number of people who commute in each day relative to the CDP population size, and the number of people who commute out each day relative to the CDP population size, the latter two of which are calculated from the output of the gravity model in Eq 23). We calculated Spearman’s rank correlation coefficient (*ρ*) to quantify these relationships. We show scatter plots of these relationships in Fig 3.

The probability that at least one autochthonous case occurred varied little with the location of introduction (Fig 2A). This is due in part to the impacts of movement as defined by the gravity model. That is, because people in smaller populations commute out frequently, they are likely to encounter a similar number of mosquitoes as people in a large population that do not commute as frequently. The 25th, 50th, and 75th percentiles for the probability of autochthonous transmission were 0.51, 0.53, and 0.55, respectively. The probability of at least one autochthonous case was negatively correlated with the population size of the location of introduction (*ρ* = −0.3023, *p* < 0.0001), which suggests that autochthonous transmission was more likely to occur when introductions appeared in locations with a smaller human population size than introductions into locations with a larger human population, although this relationship was weak. The probability of at least one autochthonous case was also positively correlated with the number of commuters (*ρ* = 0.3206, *p* < 0.0001 for inbound commuters and *ρ* = 0.3585, *p* < 0.0001 for outbound commuters), suggesting that an introduction into a location with higher numbers of commuters is more likely to lead to autochthonous transmission than those with fewer commuters, but again this relationship was weak.

The median number of cases that occurred within 100 days of introduction varied more with introductions in different locations than did the probability of at least one autochthonous case. The median number of cases occurring within 100 days of introduction fell between a minimum of 65 and a maximum of 279395, and the 25th, 50th, and 75th percentiles were 95, 122, and 207, respectively. We observed a strong negative correlation between the median number of cases occurring within 100 days of introduction and the population size of the location of introduction (*ρ* = −0.8579, *p* < 0.0001), indicating that introductions into locations with a smaller human population size typically led to larger outbreaks in the region. We also observed a strong positive correlation between the median number of cases occurring within 100 days of introduction and the relative number of commuters (*ρ* = 0.8217, *p* < 0.0001 for inbound commuters and *ρ* = 0.9320, *p* < 0.0001 for outbound commuters), which suggests that the number of commuters, and in particular, the relative number of people who commute out of the location of introduction are driving the number of cases that occur throughout the entire region following introduction.

We remark here that the impact of population size on the probability of local transmission and the median number of cases that occur within 100 days of introduction is a consequence of the assumptions associated with the use of the gravity model to approximate human movement. Due to the structure of the gravity model, there is a negative correlation between human population size and the daily number of outbound commuters. Thus, the negative correlations between population size and our two primary metrics presented above are the result of the positive correlations between the relative number of outbound commuters and our two primary metrics. If we assume that people do not move about the region (i.e., set *θ* = 0), there is no significant correlation between population size and the number of cases that occur within 100 days of introduction (*ρ* = 0.058, *p* = 0.4422), and there is a weak, but significant, positive correlation between population size and the probability of autochthonous transmission (*ρ* = 0.2696, *p* = 0.002).

### Reporting

**Fig 4:**
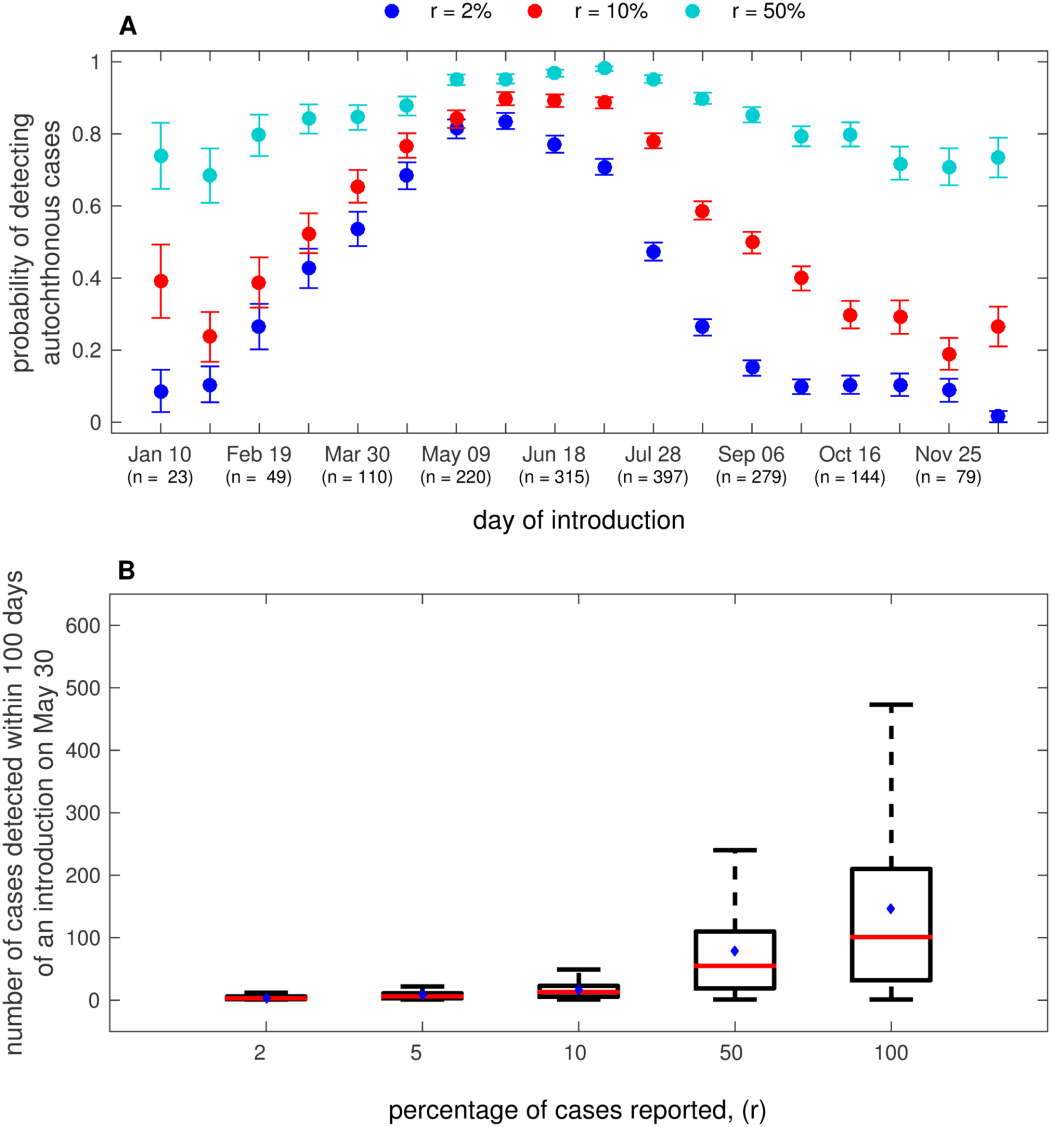
Probability of detecting local transmission at different reporting rates. (A) The probability of detecting local transmission at different reporting rates (*r*) for different days of introduction (error bars are the probability of autochthonous transmission ± the binomial standard deviation). (B) The total number of cases detected in the first 100 days following a single introduction on May 30 for different reporting rates. All other parameter values are as given in Table 1. In (A), the number in parentheses indicates the number of total simulations (out of 500 total simulations) in which at least one autochthonous case of transmission occurred. In the boxplot in (B), red lines indicate the median, blue dots indicate the mean, and the box represents the interquartile range. Whiskers indicate the interquartile range multiplied by 1.5.

The clinical presentation rate and thus the fraction of total cases that are reported will have a significant impact on whether a dengue outbreak is detected. We investigate the potential impacts of imperfect reporting rates on the probability that autochthonous transmission is detected as well as the total number of cases detected within 100 days of the initial introduction (Fig 4). We note here that, for the purpose of this study, reporting rates are synonymous with clinical presentation rates. For this analysis, we introduced a single infectious individual in the city of Miami at different times of the year, starting with January 10 and incrementing every 10 days until December 26 (as in our study of the timing of introduction), and we calculated the probability of detecting at least one autochthonous case when reporting rates (*r*) are 2%, 10%, and 50% (Fig 4A). The probability is calculated as the fraction of cases detected at each reporting rate given that autochthonous transmission actually occurred.

The probability of detecting autochthonous transmission at different reporting rates varied with the timing of introduction, largely due to the associated variation in the number of autochthonous cases (Fig 1B). However, the magnitude of that variability depended upon the reporting rate. When reporting rates were high (50%), the probability of detecting autochthonous transmission exhibited a small amount of variation with the timing of introduction, because detection of autochthonous transmission was very likely even if only a few cases occurred. At lower reporting rates, such as 2% (blue dots in Fig 4A), the probability of detecting autochthonous transmission was highest when cases were imported in late May, which corresponds to the timing of introduction that led to the highest number of cases (Fig 1B). In general, when the reporting rates were lower, the probability of detecting autochthonous transmission was strongly affected by the number of cases that occurred (compare Figs 1B and 4A). For instance, the probability of detecting autochthonous transmission was generally similar for reporting rates of 2 and 10% when introductions occurred during the first six months of the year (when outbreaks were larger, Fig 1B), whereas the probabilities differed by at least 0.2 for much of the remainder of the year (when outbreaks were smaller, Fig 1B). Although expected, this result emphasizes the influence of reporting rate on our ability to detect transmission during smaller outbreaks.

To study the impact of reporting rates on the perceived size of an outbreak, we introduced a single infectious person in the city of Miami on May 30 and compared the size of the detected outbreak. We found that the total number of cases detected could differ by as much as almost 100% at lower reporting rates. For example, the median number of cases reported when the reporting rate was 5% was fewer than 10, whereas the actual number of cases recorded for this scenario was approximately 100. At a reporting rate of only 2%, no cases may be reported even when there is an outbreak of over 100 cases. Note that this result is not surprising (e.g., 5% of 100 is 5), but it emphasizes how low reporting rates may impact the ability to detect local transmission and outbreaks, even when the outbreaks are large for the region in which they occur.

### Sensitivity Analysis

**Fig 5:**
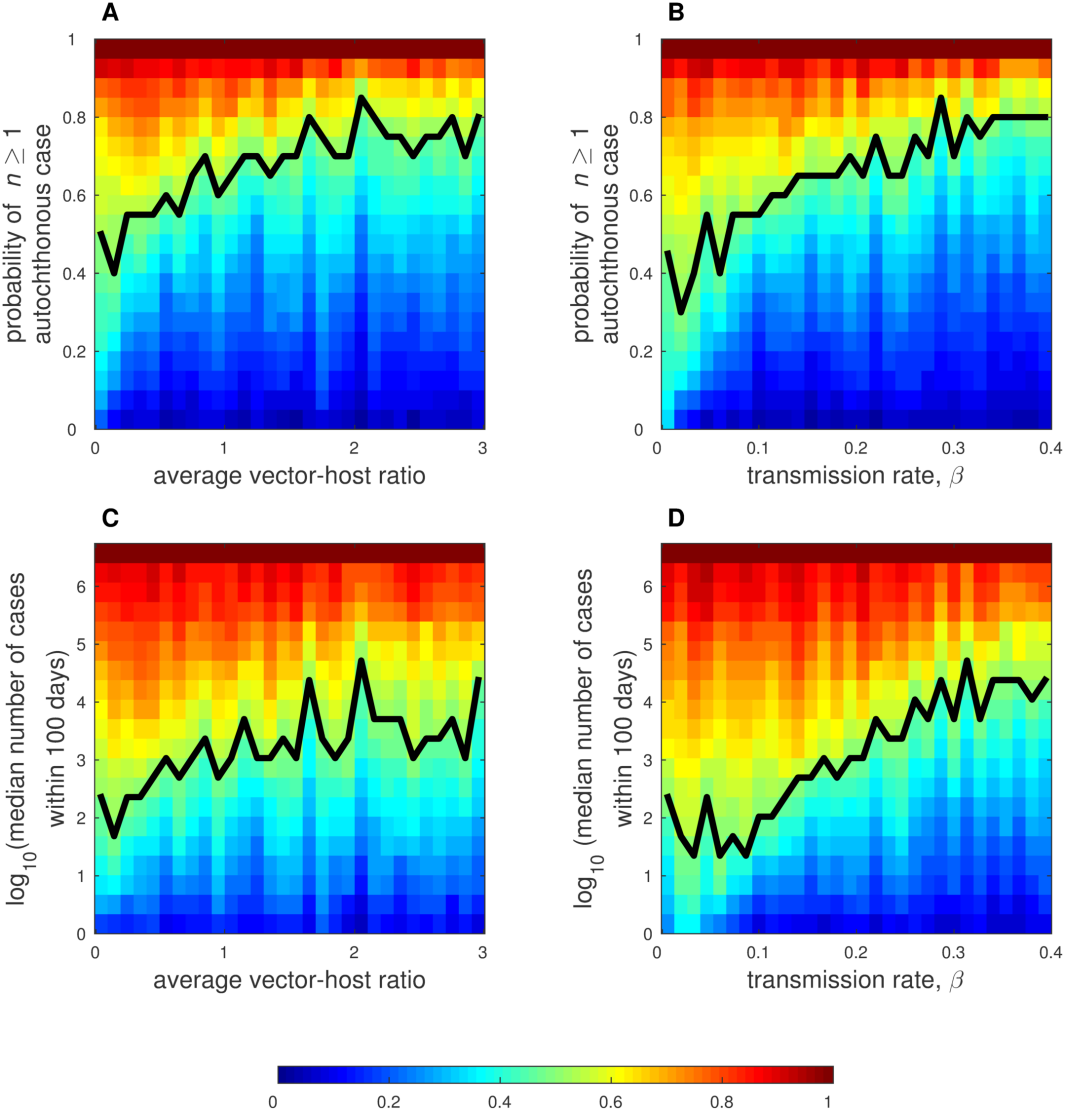
Sensitivity analysis for the average vector-host ratio and transmission rate *β*. Heat maps depict cumulative distributions of the probability of autochthonous transmission as it varies with changes in the average-vector host ratio (A) and *β* (B), and the median number of cases that occur within 100 days of introduction as it varies with changes in the average vector-host ratio (C) and *β* (D). Note that the axis for the median number of cases that occur within 100 days is on a log_10_ scale. In each heat map, the parameters on the horizontal axis are divided into 30 evenly spaced groups and the values on the vertical axis are divided into 20 evenly spaced groups. The colored rectangles present the cumulative frequency of simulations conducted with parameter values in the group on the horizontal axis that led to values of the metric in the group on the vertical axis. The solid black curve represents the median value of the metric presented on the vertical axis for each group of values on the horizontal axis (i.e. 50% of the simulations conducted with parameters in the the range of values on the horizontal axis led to values of the metric on the vertical axis higher than those at the curve). These figures were generated from 5000 total simulation sets. Each simulation set is a unique parameter combination that is run 100 times. For these simulations, the average vector-host ratio ∼ Uniform(0, 3), *β* ∼ Uniform(0, 4), and log_10_(*θ*) ∼ Uniform(−8, −4). All other parameter values are as given in Table 1.

Finally, we study the impacts on the model of changes in the average vector-host ratio and *β* by varying values for these parameters as well as the parameter *θ* in a global sensitivity analysis and observing how values of our two primary metrics (probability of autochthonous transmission and number of cases that occur within 100 days of introduction) change as values for these parameters change. In Fig 5, we present the marginal distributions of the two metrics for a range of values of the average vector-host ratio and *β*. S11 Fig shows the global distributions of the two metrics obtained in this sensitivity analysis, and S12 Fig shows $R_{0}^{T}\left(t\right)$ values on the day of introduction for each of the 5000 parameter combinations. In S1 Text, we also present results for the impacts of changes in *θ* on model output (S13 Fig).

In Fig 5 and S13 Fig, we present heat maps of the cumulative frequency of each metric. For each heat map, the parameters on the horizontal axis are divided into 30 evenly spaced groups and the values on the vertical axis are divided into 20 evenly spaced groups. The colored rectangles present the cumulative frequency of simulations conducted with parameter values in the group on the horizontal axis that led to values of the metric in the group on the vertical axis. The solid black curve represents the median of the cumulative distribution for the metric presented on the vertical axis for each group of values on the horizontal axis (i.e. 50% of the simulations conducted with parameters in the group of values on the horizontal axis led to values in the groups on the vertical axis that were higher than those at the black curve).

As the average vector-host ratio increased, the probability of autochthonous transmission increased gradually and began to saturate around an average vector-host ratio of 2 (Fig 5A). The median number of cases that occurred within 100 days of introduction also increased with the average vector-host ratio (Fig 5C). From the lowest group of values for the average vector-host ratios considered (≤ 0.1) to an average vector-host ratio near 1, the median of the cumulative distribution for the median number of cases that occurred within 100 days increased from tens of cases to hundreds of cases. As the average vector-host ratio increased beyond 1.5, the change in the median number of cases that occurred within 100 days was minimal.

As expected, increases in *β* had a greater impact than increases in the average vector-host ratio on the probability of autochthonous transmission and the median number of cases that occurred within 100 days of introduction (Fig 5B). The strong impact of this parameter on the dynamics is due in part to its role in both human-to-mosquito and mosquito-to-human transmission, which leads to a nonlinear impact on $R_{0}^{T}\left(t\right)$. As *β* increased from values close to zero to values close to 0.4, the median of the cumulative distribution for the probability of autochthonous transmission increased from approximately 0 to approximately 0.9. The values for the probability of autochthonous transmission did not saturate with increases of *β* within the group of values we considered. The median number of cases that occurred within 100 days of introduction also increased rapidly with increases in *β* (Fig 5D). The median of the cumulative distribution for the median number of cases that occurred within 100 days increased by four orders of magnitude when *β* increased from 0.1 to 0.4, which indicates that small changes in *β* can cause radically different model outcomes.

## Discussion

In this study, we developed a mathematical model to understand the potential for emergence of mosquito-borne disease in an Urbanized Area in the United States. We highlighted the utility of this model by exploring the role of timing and location of introduction in determining whether an introduction of dengue in the Miami UA will lead to autochthonous transmission and outbreaks in the context of human movement patterns. Furthermore, we utilized the model to understand the impact of reporting rates on detection and perceived outbreak size, and we tested the sensitivity of the model to parameters that are poorly understood for the Miami UA, namely the transmission rate and average vector-host ratio.

We showed that the time of year in which an imported case of dengue arrives within the Miami UA influences both the probability of autochthonous transmission and the ultimate number of locally acquired cases. Although introductions in June and July led to the highest probability of autochthonous transmission, introductions in May often led to the greatest number of cases. This result reemphasizes the need for comprehensive vector control measures to begin in late spring and early summer even if the vector population and the number of dengue cases observed at the time are low. We found that outbreaks following introductions in autumn were smaller than those following introductions in the late spring and summer. Most of the imported cases of dengue that were reported in 2010–2014 occurred in late summer and early autumn when the vector population is lower (S2 and S7 Figs). Importations in late spring and early summer have been less common. This study of the impacts of the timing of introduction also potentially assists in understanding why large outbreaks of dengue in southern Florida have been rare despite numerous imported cases.

We also found a relationship between the timing of introduction and the fraction of cases that occurred at the location of introduction. A smaller fraction of outbreaks following introduction in the spring and summer occurred within the introduction location whereas introductions in the winter led to chains of transmission that occurred almost entirely within the location of introduction. This result has the potential to inform vector control measures and encourage more efficient allocation of resources throughout the region during the months when the vector population is less abundant. Although available data does not allow us to link the outbreaks that have occurred to specific introductions in the region, the larger chains of transmission and the outbreaks that have been reported in southern Florida (which occurred in autumn) were localized to specific locations (namely, Martin County and Key West). This supports our result that the probability of an outbreak is seasonally driven.

In our effort to determine the impact of the location of introduction, we found that when dengue introductions occurred in smaller populations, the total number of autochthonous cases throughout the Miami UA was higher. This is due primarily to the higher rate of movement of people in smaller populations. That is, based on the U.S. Census commuter data and the gravity model with which we parameterized implicit human movement, people in smaller cities and cities that are farther away from larger cities traveled more about the entire region than those in large cities, and these people were potentially acting as carriers of the virus. This led to the higher probabilities of transmission and subsequent acquisition of infection by populations in the smaller CDPs and unincorporated CCDs in the Miami UA. Although this result is a consequence of the movement parameterization in the model, it could provide one explanation as to why larger outbreaks of dengue have not been observed in the heavily populated counties of the Miami UA (Miami-Dade, Broward, and Palm Beach) despite numerous introductions. In fact, the outbreaks that occurred were in counties with lower population sizes than these three (Martin County and Monroe County) [37].

The fraction of total cases of dengue that are actually reported depends on the clinical presentation rate within the population. According to a study by Bhatt and colleagues in 2013, approximately 24.6% of dengue cases are apparent, meaning that about 75.4% of dengue cases will be subclinical and thus not likely to be reported to health authorities [72]. This presentation rate and reporting will have a significant impact on whether an outbreak of dengue is detected. For instance, in 2009-2010, seroprevalence studies suggested that only about 2% of the cases that occurred in Key West, Florida presented at health care facilities and were reported [39, 62]. Investigating the impacts of reporting rates on the probability of detecting autochthonous transmission and subsequent outbreaks, we showed that at reporting rates of 2%, the probability of detecting a locally acquired case was below 0.20 when introductions occurred during the winter and fall (Fig 4A). Furthermore, we showed that at this reporting rate, a large outbreak could potentially go completely undetected (Fig 4B). This result suggests that the small chains of transmission that have occurred in the Miami UA in the past four years are possibly part of larger outbreaks. Seroprevalence studies, such as the one conducted in Key West following the 2009-2010 outbreaks [73], need to be conducted in the areas where numerous imported cases have occurred to determine whether undetected outbreaks have occurred. If seroprevalence of one or two serotypes of dengue (such as DENV1 or DENV2, the two serotypes most frequently imported in Florida [37]) is higher than assumed, this would indicate that a significant proportion of the population is at risk for secondary dengue infection should an outbreak of another serotype of dengue occur in the region in the future, which could challenge health care infrastructure.

Although we elucidated scenarios in which importation of dengue could lead to autochthonous transmission and outbreaks, we emphasize that these results were obtained for specific parameter sets. We showed that our model is sensitive to parameters whose values are not well understood, namely the dengue transmission rates and the vector-host ratio. Several studies have aimed to better understand the distribution of *Ae. aegypti* in southern Florida [42, 44, 45] and have found that *Ae. aegypti* populations tend to be associated with more developed areas within the region and that the vector populations are greater in areas closer to the Atlantic coast where the human population is also greater. Even with this knowledge, quantifying the abundance of *Ae. aegypti* and understanding the relationship between the vector population and the highly heterogeneous human population throughout the Miami UA are difficult tasks. However, developing a better understanding of the relationship between human and mosquito vector distributions as well as the potential for transmission of dengue between the two populations in this region is critical to developing a model with stronger predictive power.

In addition to a better understanding of vector distribution and transmission potential, the model presented here would benefit from a more thorough understanding of several other unknown factors that contribute to heterogeneity of risk of infection throughout the Miami UA, such as human movement and activity in the region and heterogeneity in human contact with vectors. Risk of infection in our model is associated primarily with population size and movement according to a parameterization of the gravity model obtained using U.S. Census commuter data. Movement of humans is strongly associated with the spread of dengue throughout a region [27, 28], but movement is more complex than that captured by the gravity model and the data used to parameterize it. For instance, travel within a CDP, travel beyond that of daily commuting, and recreational travel are all likely to influence risk of transmission. To our knowledge, this information is not available for the Miami UA, but studies have been conducted in some dengue endemic regions such as Iquitos, Peru to better understand heterogeneity in human movement and how that heterogeneity potentially impacts dengue risk [74]. Similar studies for the Miami UA as well as other areas within the U.S. where arbovirus introduction is likely to lead to an outbreak would allow for investigation of the impacts on local transmission and outbreak of different types of human movement.

This model also does not account for any spatiotemporal heterogeneity of the human population or mosquito behavior. Human-mosquito contact within each of the subpopulations of the Miami UA is assumed to be well-mixed in this model, but this is not likely the case for all locations. Some locations potentially have varying degrees of assortative mixing that may depend upon a number of factors, including daily activities and socioeconomic status. While general socioeconomic measures are available for the CDPs and CCDs from the U.S. Census [75], this information is not as easily obtainable for subdivisions of the CDPs and CCDs. Furthermore, incorporating these measures into the model, either within or across locations, would require an established relationship between socioeconomic status and human contact with vectors within the Miami UA. To our knowledge, a study of this relationship has not been conducted for the Miami UA, but studies to begin to quantify this relationship for dengue and West Nile have been conducted in other locations within the U.S. such as Baltimore, Maryland and Suffolk County, New York, among other places [76–78]. While the relationships derived from these studies may not apply specifically to dengue in the Miami UA, they could be incorporated in the model as an *a priori* assessment of the potential impact of socioeconomic heterogeneity on disease spread and outbreaks. Heterogeneity in vector-host ratios and contact with vectors could be another reason why introductions of dengue in larger populations have not yet led to outbreaks while outbreaks have occurred in smaller populations (S1 Fig, [37]). In addition to the influences of spatial heterogeneity, temporal variations in human exposure to mosquitoes and mosquito feeding habits should be considered. Changes in precipitation and temperature that occur across seasons are likely to influence human activity, and there is evidence that *Ae. aegypti* biting rates are influenced by temperature [79], although the influence of these temporally varying aspects on dengue transmission in the Miami UA are not well understood.

The structure of our model follows a common framework for epidemiological models and is parameterized with publicly available U.S. Census population and daily commute data [51, 55], making this model adaptable to other regions of the United States. Although it is designed to study mosquito-borne disease spread throughout urbanized areas, it can also be utilized to study other diseases by adapting the basic epidemiological structure, and it can be parameterized at the level of counties, CCDs, or CDPs for any region where similar data is available. For example, this model could be adapted to study two other potential threats to the Miami UA and other metropolitan areas in the U.S. such as New Orleans, Houston, and New York City: chikungunya and Zika virus. Chikungunya became established in the western hemisphere in 2014 and imported cases were detected in nearly all U.S. States. Over 400 imported cases were detected in Florida in 2014, and the majority of those were in southern Florida [37] (S14 Fig). New York reported the most imported cases of chikungunya of any state within the United States (801) and although no locally transmitted cases were reported there, one of the major vectors of chikungunya and dengue, *Ae. albopictus*, is present throughout the region [78, 80]. Zika virus was first detected in the Western Hemisphere in 2015 and is rapidly spreading throughout Central and South America [7]. As of mid-June 2016, 755 travel-related cases of Zika virus have been reported in the United States, 147 of which were reported in Florida (S15 Fig), but no autochthonous cases due to mosquito-human contact have been reported yet [37, 81]. While modeling studies have been conducted to study the potential spread of chikungunya virus throughout the Americas, including cities within the United States [10–12], no studies have examined the potential for introduction and emergence of dengue, chikungunya, or Zika virus within U.S. cities with the detail afforded by the model presented here.

## Supporting Information

S1 TextSupplementary Text.We describe the acquisition of U.S. Census data, the fitting of the vector dynamics and gravity model to data, the results of sensitivity analysis for the parameter *θ*, the calculation of $R_{0}^{T}\left(i,t\right)$, and complexities of parameter selection.(PDF)

S1 Fig Total dengue cases in Florida 2010–2016. Total cases of dengue imported (A) and locally acquired (B) in the Miami UA and Florida. Broward, Miami-Dade, and Palm Beach counties comprise the majority of the Miami UA. Martin County, part of which is considered to be within the Miami UA, is located just north of Palm Beach county. Monroe county includes the Florida Keys and is located west and southwest of the Miami UA. Data presented here were obtained from [37]. Note that 2016 numbers are as of June 15, 2016.
(PDF)

S2 Fig Time series of dengue cases in Florida 2010–2015. Time series of the number of imported (A,C) and locally acquired (B,D) cases of dengue in the Miami UA (Broward, Miami-Dade, and Palm Beach counties, A,B) and the remainder of Florida (C,D). Data presented in these figures were obtained from [37]. Each curve in the figure represents a different year (2010–2015). Note that the time associated with cases is the time at which Florida Department of Health reported the cases.
(PDF)

S3 FigThe time-varying value of $R_{0}^{T}\left(t\right)$ (see Eq 22) in one CDP of introduction.
$R_{0}^{T}\left(t\right)$ (not taking into account the influence of human movement) at different times of the year when the average vector-host ratio is 1. Note that this calculation assumes that the vector-host ratio remains the same from the day of introduction forward and is thus an approximation of the value of *R*_0_ on the day in which an imported case is introduced. The dashed black line indicates the epidemiological threshold value of $R_{0}^{T}\left(t\right)=1$. This $R_{0}^{T}\left(t\right)$ value is calculated with the parameters listed in Table 1 of the main text.(PDF)

S4 Fig (Top) Location of the Miami UA in Florida. (Bottom) Population size (2010) of the cities, towns, villages, CDPs, and CCDs within the Miami UA. The counties in the Miami UA are, from top to bottom, Martin, Palm Beach, Broward, and Miami-Dade. The map included in this figure was obtained from U.S. Census Bureau TIGER/LINE^®^Shapefiles [71].
(PDF)

S5 Fig (A) Residual values of gravity model predictions (actual commuter flow—model estimate). (B) Boxplot of residual values (excluding outliers). In the boxplot, the red line represents the median value and the box represents the interquartile range.
(PDF)

S6 Fig Location of CDC Light Traps in Miami-Dade county. The map included in this figure was obtained from U.S. Census Bureau TIGER/LINE^®^Shapefiles [71].
(PDF)

S7 Fig *Ae. aegypti* population counts from CDC light traps from 2010–2013. Blue dots are weekly counts and the solid black line is Eq S4 fit to each trap using the data presented in each figure.
(PDF)

S8 Fig Deterministic dynamics of the *Ae. aegypti* population throughout a single year for different values of the average vector-host ratio. The curves represent the vector-host ratio as it changes throughout the year with fluctuations in the vector population. Each curve represents a deterministic simulation with a different value for the average vector-host ratio. In the main text, the average vector-host ratio is 1, which is indicated by the black curve in this figure.
(PDF)

S9 Fig Relationship between *β* and the average vector-host ratio in plausible parameter sets. Scatter plot of 3000 combinations of values of *β* and the average vector-host ratio utilized in simulations of dengue introduction in a single homogeneous population of size 38000. Light blue circles represent all combinations of the two parameters. Dark black circles represent the values of the two parameters that were deemed plausible (i.e. led to a maximum number of cases from 100 simulations between 170–340). The pink star represents the combination of the two parameters utilized as the default values in this study. For more information, see S1 Text.
(PDF)

S10 Fig Boxplots of Plausible Parameter sets. (A) The maximum number of cases from 100 simulations of each parameter set. The red squares denote the upper (340) and lower (170) bounds on the maximum number of infections required for a parameter set to be deemed plausible. (B) *β*. (C) *θ* (log scale), and (D) The average vector-host ratio. For panels (B-D), the red star represents the default value chosen for that parameter from one plausible parameter set. The red line represents the median, and the box encases the Interquartile range (IQR). The whiskers indicate 1.5×IQR.
(PDF)

S11 Fig Distribution of metrics for all 5000 simulations utilized in the global sensitivity analysis. (A) The relative frequency of the probability of at least one autochthonous case. (B) The relative frequency of the median number of cases that occurred within 100 days of introduction (on a log_10_ scale). For these simulations, the average vector-host ratio was chosen from a uniform distribution with minimum 0 and maximum 3, *β* ∼ uniform(0, 0.4), and log_10_(*θ*) ∼ uniform(-8, -4). All other parameter values are as given in Table 1 of the main text.
(PDF)

S12 FigDistribution of $R_{0}^{T}\left(t\right)$ (see Eq 22) for a single population on May 30 for all 5000 simulations utilized in the global sensitivity analysis.The relative frequency of $R_{0}^{T}\left(t\right)$ on May 30. For these simulations, the average vector-host ratio was chosen from a uniform distribution with minimum 0 and maximum 3, *β* ∼ uniform(0, 0.4), and log_10_(*θ*) ∼ uniform(-8, -4). All other parameter values are as given in Table 1 of the main text.(PDF)

S13 Fig Sensitivity analysis for the parameter *θ*. Heat maps depict how the probability of autochthonous transmission (A) changes with changes in *θ* and how the median number of cases that occurred within 100 days of introduction (B) changes with changes in *θ*. Note that the axis for the median number of cases that occur within 100 days is on a *log*_10_ scale. In each heat map, the parameters on the horizontal axis are divided into 30 evenly spaced groups and the values on the vertical axis are divided into 20 evenly spaced groups. The colored rectangles represent the cumulative frequency of simulations conducted with parameter values in the group on the horizontal axis that led to values of the metric in the group on the vertical axis. The solid black curve represents the median of the cumulative distribution for the metric presented on the vertical axis for each group of values on the horizontal axis. These figures were generated from 5000 total simulation sets. Each simulation set is a unique parameter combination that is run 100 times. For these simulations, the average vector-host ratio ∼ uniform(0, 3), *β* ∼ uniform(0, 4), and log_10_(*θ*) ∼ uniform(−8, −4). All other parameter values are as given in Table 1 of the main text.
(PDF)

S14 Fig Total cases of chikungunya in Florida 2014–2016. Imported (A) and locally acquired (B) chikungunya cases in the Miami UA and Florida. Broward, Miami-Dade, and Palm Beach counties are part of the Miami UA. Martin County, part of which is considered to be within the Miami UA, is just north of Palm Beach County. Monroe county includes the Florida Keys and is west and southwest of the Miami UA. Data presented in these figures were aggregated from [37]. Note that 2016 numbers are as of June 15, 2016.
(PDF)

S15 Fig Total cases of Zika virus in Florida 2016. Imported Zika virus cases in the Miami UA and Florida. Broward, Miami-Dade, and Palm Beach counties are part of the Miami UA. Martin County, part of which is considered to be within the Miami UA, is just north of Palm Beach County. Monroe county includes the Florida Keys and is west and southwest of the Miami UA. Data presented in these figures were aggregated from [37]. Note that numbers are as of June 15, 2016.
(PDF)

## References

[1] Gubler DJ. Resurgent vector-borne diseases as a global health problem. Emerging Infectious Diseases. 1998;4(3):442–50. 10.3201/eid0403.980326 9716967 PMC2640300

[2] Gubler DJ. Vector-borne diseases. Revue Scientifique et Technique (International Office of Epizootics). 2009 8;28(2):583–8. 20128467 10.20506/rst.28.2.1904

[3] Tatem AJ, Huang Z, Das A, Qi Q, Roth J, Qiu Y. Air travel and vector-borne disease movement. Parasitology. 2012 12;139(14):1816–30. 10.1017/S0031182012000352 22444826

[4] Mackenzie JS, Gubler DJ, Petersen LR. Emerging flaviviruses: the spread and resurgence of Japanese encephalitis, West Nile and dengue viruses. Nature Medicine. 2004 12;10(12):S98–109. 10.1038/nm1144 15577938

[5] Nasci RS. Movement of Chikungunya Virus into the Western Hemisphere. Emerging Infectious Diseases. 2014 8;20(8):1394–1395. 10.3201/eid2008.140333 25061832 PMC4111178

[6] Rezza G. Dengue and chikungunya: long-distance spread and outbreaks in naïve areas. Pathogens and Global Health. 2014 12; 108(8): 349–55. 10.1179/2047773214Y.0000000163 25491436 PMC4394667

[7] Hennessey M, Fisher M, Staples JE. Zika Virus Spreads to New Areas-Region of the Americas, May 2015-January 2016. Morbidity & Mortality Weekly Report. 2016;65(3):55–58. Available: http://www.cdc.gov/mmwr/volumes/65/wr/mm6503e1.htm. 10.15585/mmwr.mm6503e1 26820163

[8] Anderson RM, May RM. Infectious Disease of Humans. Oxford: Oxford University Press; 1991.

[9] Christofferson RC, Mores CN, Wearing HJ. Characterizing the likelihood of dengue emergence and detection in naive populations. Parasites & Vectors. 2014 6;7(1):282 10.1186/1756-3305-7-282 24957139 PMC4082489

[10] Ruiz-Moreno D, Vargas IS, Olson KE, Harrington LC. Modeling dynamic introduction of chikungunya virus in the United States. PLoS Neglected Tropical Diseases. 2012 1;6(11):e1918 10.1371/journal.pntd.0001918 23209859 PMC3510155

[11] Cauchemez S, Ledrans M, Poletto C. Local and regional spread of chikungunya fever in the Americas. Euro Surveill. 2014;19(28):20854 10.2807/1560-7917.ES2014.19.28.20854 25060573 PMC4340072

[12] Johansson MA, Powers AM, Pesik N, Cohen NJ, Staples JE. Nowcasting the spread of chikungunya virus in the Americas. PLoS ONE. 2014 1;9(8):e104915 10.1371/journal.pone.0104915 25111394 PMC4128737

[13] Poletti P, Messeri G, Ajelli M, Vallorani R, Rizzo C, Merler S. Transmission potential of chikungunya virus and control measures: The case of Italy. PLoS ONE. 2011;6(5). 10.1371/journal.pone.0018860 PMC3086881 21559329

[14] Lourenço J, Recker M. The 2012 Madeira dengue outbreak: epidemiological determinants and future epidemic potential. PLoS Neglected Tropical Diseases. 2014;8(8):e3083 10.1371/journal.pntd.0003083 25144749 PMC4140668

[15] Feldstein LR, Brownstein JS, Brady OJ, Hay SI, Johansson MA. Dengue on islands: A Bayesian approach to understanding the global ecology of dengue viruses. Transactions of the Royal Society of Tropical Medicine and Hygiene. 2015;p. 1–10. 25771261 10.1093/trstmh/trv012 PMC4401210

[16] Gubler DJ. Epidemic dengue/dengue hemorrhagic fever as a public health, social and economic problem in the 21st century. Trends in Microbiology. 2002 2;10(2):100–3. 10.1016/S0966-842X(01)02288-0 11827812

[17] Suk JE, Semenza JC. From global to local: vector-borne disease in an interconnected world. European Journal of Public Health. 2014 8;24(4):531–2. 10.1093/eurpub/cku041 25063828

[18] Semenza JC, Sudre B, Miniota J, Rossi M, Hu W, Kossowsky D, et al International Dispersal of Dengue through Air Travel: Importation Risk for Europe. PLoS Neglected Tropical Diseases. 2014 12;8(12):e3278 10.1371/journal.pntd.0003278 25474491 PMC4256202

[19] Johansson MA, Arana-Vizcarrondo N, Biggerstaff BJ, Staples JE, Gallagher N, Marano N. On the treatment of airline travelers in mathematical models. PLoS ONE. 2011 1;6(7):e22151 10.1371/journal.pone.0022151 21799782 PMC3143116

[20] Johansson MA, Arana-Vizcarrondo N, Biggerstaff BJ, Gallagher N, Marano N, Staples JE. Assessing the risk of international spread of yellow fever virus: a mathematical analysis of an urban outbreak in Asuncion, 2008. The American Journal of Tropical Medicine and Hygiene. 2012 2;86(2):349–58. 10.4269/ajtmh.2012.11-0432 22302873 PMC3269406

[21] Benedict MQ, Levine RS, Hawley WA, Lounibos LP. Spread of the tiger: global risk of invasion by the mosquito *Aedes albopictus*. Vector-borne and Zoonotic Diseases. 2007 1;7(1):76–85. 10.1089/vbz.2006.0562 17417960 PMC2212601

[22] Knudsen AB. Global distribution and continuing spread of *Aedes albopictus*. Parassitologia. 1995 12;37(2–3):91–7. 8778670

[23] Gubler DJ. Prevention and Control of *Aedes aegypti*-borne Diseases: Lesson Learned from Past Successes and Failures. Asian-Pacific Journal of Molecular Biology and Biotechnology. 2011; 19(3): 111–114.

[24] Guagliardo SA, Barboza JL, Morrison AC, Astete H, Vazquez-Prokopec G, Kitron U. Patterns of Geographic Expansion of *Aedes aegypti* in the Peruvian Amazon. PLoS Neglected Tropical Diseases. 2014 8;8(8):e3033 10.1371/journal.pntd.0003033 25101786 PMC4125293

[25] Guagliardo SA, Morrison AC, Luis Barboza J, Wesson DM, Ponnusamy L, Astete H, et al Evidence for *Aedes aegypti* (Diptera: Culicidae) oviposition on boats in the Peruvian Amazon. Journal of Medical Entomology. 2015;52(4):726–729. 10.1093/jme/tjv048 26335482 PMC4592347

[26] Guagliardo SA, Morrison AC, Barboza JL, Requena E, Astete H, Vazquez-Prokopec G, et al River boats contribute to the regional spread of the dengue vector *Aedes aegypti* in the Peruvian Amazon. PLoS Neglected Tropical Diseases. 2015; 9(4):e0003648 10.1371/journal.pntd.0003648 25860352 PMC4393238

[27] Stoddard ST, Forshey BM, Morrison AC, Paz-Soldan VA, Vazquez-Prokopec GM, Astete H, et al House-to-house human movement drives dengue virus transmission. Proceedings of the National Academy of Sciences of the United States of America. 2013 1;110(3):994–9. 10.1073/pnas.1213349110 23277539 PMC3549073

[28] Reiner RC, Stoddard ST, Scott TW. Socially structured human movement shapes dengue transmission despite the diffusive effect of mosquito dispersal. Epidemics. 2014 3;6:30–36. 10.1016/j.epidem.2013.12.003 24593919 PMC3971836

[29] Adams B, Kapan DD. Man bites mosquito: understanding the contribution of human movement to vector-borne disease dynamics. PloS ONE. 2009 1;4(8):e6763 10.1371/journal.pone.0006763 19707544 PMC2727792

[30] Erlander S, Stewart NF. The gravity model in transportation analysis: theory and extensions. Utrecht, The Netherlands: VSP; 1990.

[31] Xia Y, Bjørnstad ON, Grenfell BT. Measles metapopulation dynamics: a gravity model for epidemiological coupling and dynamics. The American Naturalist. 2004 8;164(2):267–81. 10.1086/422341 15278849

[32] Viboud C, Bjø rnstad ON, Smith DL, Simonsen L, Miller MA, Grenfell BT. Synchrony, waves, and spatial hierarchies in the spread of influenza. Science (New York, NY). 2006 4;312(5772):447–51. 10.1126/science.1125237 16574822

[33] Bhoomiboonchoo P, Gibbons RV, Huang A, Yoon IK, Buddhari D, Nisalak A, et al The spatial dynamics of dengue virus in kamphaeng phet, Thailand. PLoS Neglected Tropical Diseases. 2014 9;8(9):e3138 10.1371/journal.pntd.0003138 25211127 PMC4161352

[34] Wesolowski A, Qureshi T, Boni MF, Sundsø y PR, Johansson MA, Rasheed SB, et al Impact of human mobility on the emergence of dengue epidemics in Pakistan. Proceedings of the National Academy of Sciences. 2015;p. 201504964. 10.1073/pnas.1504964112 PMC4586847 26351662

[35] World Health Organization. Dengue and severe dengue—fact sheet; 2015. Available: http://www.who.int/mediacentre/factsheets/fs117/en/.

[36] Rey J. Dengue in Florida (USA). Insects. 2014 12;5(4):991–1000. 10.3390/insects5040991 26462955 PMC4592614

[37] Florida Department of Health. Weekly Surveillance Information, Mosquito-borne Diseases; 2015. Available: http://www.floridahealth.gov/diseases-and-conditions/mosquito-borne-diseases/archive-surveillance.html.

[38] U. S. Geological Survey. USGS Disease Maps; 2015. Available from: http://diseasemaps.usgs.gov/.

[39] Centers for Disease Control and Prevention (CDC). Locally Acquired Dengue x2014; Key West, Florida, 2009–2010. Morbidity & Mortality Weekly Report. 2010;59(19):577–581. Available: http://www.medscape.com/viewarticle/722515. 20489680

[40] O’Meara GF, Evans LF, Gettman AD, Cuda JP. Spread of *Aedes albopictus* and Decline of *Ae. aegypti* (Diptera: Culicidae) in Florida. Journal of Medical Entomology. 1995;32(4):554–562. 10.1093/jmedent/32.4.554 7650719

[41] Juliano SA, O’Meara GF, Morrill JR, Cutwa MM. Desiccation and thermal tolerance of eggs and the coexistence of competing mosquitoes. Oecologia. 2002;130(3):458–469. 10.1007/s004420100811 20871747 PMC2944657

[42] Braks MAH, Honório NA, Lourenço-De-Oliveira R, Juliano SA, Lounibos LP. Convergent habitat segregation of *Aedes aegypti* and *Aedes albopictus* (Diptera: Culicidae) in southeastern Brazil and Florida. Journal of Medical Entomology. 2003 11;40(6):785–94. 10.1603/0022-2585-40.6.785 14765654

[43] Juliano SA, Lounibos LP, O’Meara GF. A field test for competitive effects of *Aedes albopictus* on *A. aegypti* in South Florida: differences between sites of coexistence and exclusion? Oecologia. 2004 5;139(4):583–93. 10.1007/s00442-004-1532-4 15024640 PMC1906877

[44] Rey J, Nishimura N. Habitat segregation of mosquito arbovirus vectors in South Florida. Journal of Medical Entomology. 2006;43(6):1134–1141. 10.1093/jmedent/43.6.1134 17162945 PMC1820833

[45] Reiskind MH, Lounibos LP. Spatial and temporal patterns of abundance of *Aedes aegypti* L. (*Stegomyia aegypti*) and *Aedes albopictus* (Skuse) [*Stegomyia albopictus* (Skuse)] in southern Florida. Medical and Veterinary Entomology. 2013 12;27(4):421–9. 10.1111/mve.12000 23278304

[46] The Bureau of Transportation Statistics. State Transportation Statistics 2015; 2015. Available: http://www.rita.dot.gov/bts/sites/rita.dot.gov.bts/files/publications/state_transportation_statistics/state_transportation_statistics_2015/chapter-4/table4_6.

[47] Miami-Dade Aviation Department. 2013 Annual Report of the Miami International Airport; 2013. Available from: http://www.miami-airport.com/annual_report.asp.

[48] U.S. Department of Transportation Bureau of Transportation Statistics. Cruise Ship Statistics; 2012. Available from: http://www.rita.dot.gov/bts/data_and_statistics/by_subject/passenger.html.

[49] Abbey H. An examination of the Reed-Frost theory of epidemics. Human Biology. 1952;24(3):201–233. 12990130

[50] Heesterbeek JAP, Roberts MG. The type-reproduction number T in models for infectious disease control. Mathematical Biosciences. 2007 3;206(1):3–10. 10.1016/j.mbs.2004.10.013 16529777

[51] U. S. Census Bureau. OnTheMap; 2010. Available: http://onthemap.ces.census.gov/.

[52] Muir LE, Kay BH. *Aedes aegypti* survival and dispersal estimated by mark-release-recapture in northern Australia. The American Journal of Tropical Medicine and Hygiene. 1998;58(3):277–82. 9546403 10.4269/ajtmh.1998.58.277

[53] Harrington LC, Scott TW, Lerdthusnee K, Coleman RC, Costero A, Clark GG, et al Dispersal of the dengue vector *Aedes aegypti* within and between rural communities. American Journal of Tropical Medicine and Hygiene. 2005;72(2):209–220. 15741559

[54] Trpis M, Hausermann W. Dispersal and other population parameters of *Aedes aegypti* in an African village and their possible significance in epidemiology of vector-borne-diseases. The American Journal of Tropical Medicine and Hygiene. 1986;35(6):1263–1279. 3789275 10.4269/ajtmh.1986.35.1263

[55] U. S. Census Bureau. 2010 Census Population and Housing Tables (CPH-Ts); 2010. Available: http://www.census.gov/population/www/cen2010/cph-t/cph-t-9.html.

[56] Carrington LB, Armijos MV, Lambrechts L, Scott TW. Fluctuations at a low mean temperature accelerate dengue virus transmission by *Aedes aegypti*. PLoS Neglected Tropical Diseases. 2013;7(4). 10.1371/journal.pntd.0002190 PMC3636080 23638208

[57] Fouque F, Carinci R, Gaborit P, Issaly J, Bicout DJ, Sabatier P. *Aedes aegypti* survival and dengue transmission patterns in French Guiana. Journal of Vector Ecology. 2006 12;31(2):390–9. 10.3376/1081-1710(2006)31[390:AASADT]2.0.CO;2 17249358

[58] Rudolph KE, Lessler J, Moloney RM, Kmush B, Cummings DAT. Incubation periods of mosquito-borne viral infections: A systematic review. The American Journal of Tropical Medicine and Hygiene. 2014 3; 90(5):882–891. 10.4269/ajtmh.13-0403 24639305 PMC4015582

[59] Gubler DJ, Suharyono W, Tan R, Abidin M, Sie A. Viraemia in patients with naturally acquired dengue infection. Bulletin of the World Health Organization. 1981;59(4):623–630. 6976230 PMC2396101

[60] Vaughn DW, Green S, Kalayanarooj S, Innis BL, Nimmannitya S, Suntayakorn S, et al Dengue viremia titer, antibody response pattern, and virus serotype correlate with disease severity. The Journal of Infectious Diseases. 2000;181(1):2–9. 10.1086/315215 10608744

[61] Drake JM, Kaul RB, Alexander LW, O’Regan SM, Kramer AM, Pulliam JT, et al Ebola cases and health system demand in Liberia. PLOS Biology. 2015;13(1):e1002056 10.1371/journal.pbio.1002056 25585384 PMC4293091

[62] Adalja AA, Sell TK, Bouri N, Franco C. Lessons learned during dengue outbreaks in the United States, 2001–2011. Emerging Infectious Diseases. 2012;18(4):608–614. 10.3201/eid1804.110968 22469195 PMC3309700

[63] Ross R. The prevention of malaria. London, UK: John Murray; 1910.

[64] Ross R. Some quantitative studies in epidemiology. Nature. 1911;87:466–467. 10.1038/087466a0

[65] Macdonald G. The analysis of equilibrium in malaria. Tropical Disease Bulletin. 1952;49(9):813–829. 12995455

[66] de Jong MCM, Diekmann O, Heesterbeek H. How does transmission of infection depend on population size? Epidemice Models. Publication of the Newton Institute 1995:84–94.

[67] Bacaër N. Approximation of the basic reproduction number *R*_0_ for vector-borne diseases with a periodic vector population. Bulletin of Mathematical Biology. 2007 4;69(3):1067–91. 10.1007/s11538-006-9166-9 17265121

[68] Altizer S, Dobson A, Hosseini P, Hudson P, Pascual M, Rohani P. Seasonality and the dynamics of infectious diseases. Ecology Letters. 2006;9(4):467–484. 10.1111/j.1461-0248.2005.00879.x 16623732

[69] Hufnagel L, Brockmann D, Geisel T. Forecast and control of epidemics in a globalized world. Proceedings of the National Academy of Sciences of the United States of America. 2004;101(42):15124–15129. 10.1073/pnas.0308344101 15477600 PMC524041

[70] Read JM, Keeling MJ. Disease evolution on networks: the role of contact structure. Proceedings Biological sciences / The Royal Society. 2003 4;270(1516):699–708. 10.1098/rspb.2002.2305 12713743 PMC1691304

[71] U. S. Census Bureau. Tiger/Line Shapefiles and Tiger/Line Files; 2010. Available: https://www.census.gov/geo/maps-data/data/tiger-line.html

[72] Bhatt S, Gething PW, Brady OJ, Messina JP, Farlow AW, Moyes CL, et al The global distribution and burden of dengue. Nature. 2013 4;496(7446):504–7. 10.1038/nature12060 23563266 PMC3651993

[73] Messenger AM, Barr KL, Weppelmann TA, Barnes AN, Anderson BD, Okech BA, et al Serological evidence of ongoing transmission of dengue virus in permanent residents of Key West, Florida. Vector-borne and Zoonotic Diseases. 2014 11;14(11):783–7. 10.1089/vbz.2014.1665 25409268

[74] Vazquez-Prokopec GM, Bisanzio D, Stoddard ST, Paz-Soldan V, Morrison AC, Elder JP, et al Using GPS Technology to quantify human mobility, dynamic contacts and infectious disease dynamics in a resource-poor urban environment. PLoS One. 2013;8(4):1–10. 10.1371/journal.pone.0058802 PMC3620113 23577059

[75] U. S. Census Bureau. American FactFinder; 2010. Available from: http://factfinder.census.gov/faces/nav/jsf/pages/index.xhtml.

[76] Dowling Z, Ladeau SL, Armbruster P, Biehler D, Leisnham PT. Socioeconomic status affects mosquito (Diptera: Culicidae) larval habitat type availability and infestation level. BioOne 2013;50(4):764–772. 10.1603/me12250 23926774

[77] Becker B, Leisnham PT, Ladeau SL. A tale of two city blocks: Differences in immature and adult mosquito abundances between socioeconomically different urban blocks in Baltimore (Maryland, USA). International Journal of Environmental Research and Public Health. 2014 1;11(3):3256–70. 10.3390/ijerph110303256 24651396 PMC3987033

[78] Rochlin I, Ninivaggi DV, Hutchinson ML, Farajollahi A. Climate change and range expansion of the Asian tiger mosquito (*Aedes albopictus*) in Northeastern USA: Implications for public health practitioners. PLoS ONE. 2013;8(4):1–9. 10.1371/journal.pone.0060874 PMC3614918 23565282

[79] Scott TW, Amerasinghe PH, Morrison AC, Lorenz LH, Clark GG, Strickman D, et al Longitudinal studies of *Aedes aegypti* (Diptera: Culicidae) in Thailand and Puerto Rico: blood feeding frequency. Journal of Medical Entomology. 2000 1;37(1):89–101. 15218911 10.1603/0022-2585-37.1.89

[80] U. S. Centers for Disease Control and Prevention. Chikungunya virus in the United States, 2014; 2014. Available: http://www.cdc.gov/chikungunya/geo/united-states-2014.html.

[81] U. S. Centers for Disease Control and Prevention. Zika virus disease in the United States 2015-2016, 2016; 2016. Available: http://www.cdc.gov/zika/geo/united-states.html.

