# Supplementary material for: Modeling Mosquito-Borne Disease Spread in U.S. Urbanized Areas: The Case of Dengue in Miami
Source: PLoS One. 2016 Aug 17;11(8):e0161365. doi: 10.1371/journal.pone.0161365 (PMC4988691; doi:10.1371/journal.pone.0161365)
Supplement: S1 Text — We describe the acquisition of U.S. Census data, the fitting of the vector dynamics and gravity model to data, the results of sensitivity analysis for the parameter θ, the calculation of R0T(i,t), and complexities of parameter selection. (PDF) [file pone.0161365.s001.pdf]

Modeling mosquito-borne disease spread in U.S. Urbanized Areas: the case of dengue in Miami

Michael A. Robert[1,2,*], Rebecca C. Christofferson[3], Noah J.B. Silva[1], Chalmers Vasquez[4], Christopher N. Mores[3], Helen J. Wearing[1,2]

1 Department of Biology, University of New Mexico, Albuquerque, NM, USA

2 Department of Mathematics and Statistics, University of New Mexico, Albuquerque, NM, USA

3 Department of Pathobiological Sciences, Louisiana State University, Baton Rouge, LA, USA

4 Miami-Dade County Mosquito Control Division, Miami, FL, USA

Supplementary Text

Acquisition of census data

U.S. Census population data

We parameterized the population structure of our model utilizing 2010 U.S. Census data of Urbanized Areas within the United States [1] (www.census.gov/population/www/cen2010/cph-t/cph-t-9.html). According to the U.S. Census, Urbanized Areas are divided into counties, counties are divided into Census County Divisions (CCDs), and CCDs are divided into Census Designated Places (CDPs). CDPs are cities, towns, villages, or neighborhoods. Some populations within some CCDs of the Miami UA are not included in CDPs, and we include these populations within a separate division of each particular CCD, the unincorporated area of the CCD. With the 161 given CDPs and the 25 unincorporated areas of CCDs, there are 186 total subpopulations of the Miami UA. Note that in the data presented in [1], some CDPs are found in more than one CCD. For the purpose of this study, we considered populations only at the CDP level, so any CDP that is found in more than one CCD in the U.S. Census data is included in our model structure as a single CDP.

U.S. Census commuter data

We obtained daily commute data from the U.S. Census OnTheMap tool found at http://onthemap.ces.census.gov [2]. To collect this data, we utilized the 'Text-Only' tool on the front page of the OnTheMap website. From here, we obtained data for each of the 161 CDPs individually. Note that because unincorporated areas of CCDs are not included explicitly in the OnTheMap commuter data, we can not obtain commuter flows for these populations for the parameterization of the gravity model. To access commuter flow for each location, we followed the following steps:

1. In the search box, input the CDP name, choose Places (Cities, CDPs, etc.) from the drop down menu, and click 'Go'.
2. In the list of places on the following page, choose the CDP of interest.
3. On the following page, select 'Home' as the Home/Work Area.
4. Select 'Destination' and 'Places (cities, CDPs, etc.)' as the Analysis Type.
5. Select 2010 for the year.
6. Select 'All Jobs' for the Job Type.
7. Select Run Analysis.
8. On the next page, under Settings - Number of Results, choose 'All' and select 'Update'.

The resulting page lists Job Counts by Place for the CDP that was chosen. The number of workers who commute to each place is given along with the total percentage of workers in the CDP chosen that work in each location listed. Note that the entire population in the CDP chosen is not included in the number of workers listed for the CDP. Also note that the data presented is the location of jobs of workers in the CDP chosen regardless of whether the work location is within the Miami UA. For this study, we extracted only the work locations within the Miami UA. We repeated this process for each of the CDPs within the Miami UA, and utilized this data to parameterize the gravity model to approximate human movement.

Parameter estimation for gravity model

The general gravity model that we utilized for this study is given by

$$\hat{m}_{i,j} = \sum_{j} \frac{(N_i)^a (N_j)^b}{(d_{i,j})^c} \tag{1}$$

where $\hat{m}_{i,j}$ denotes an estimate of the number of daily commuters from location i to location j, N_i and N_j represent the population size of locations i (the donor population) and j (the recipient population), respectively, and $d_{i,j}$ is the Haversine distance between locations i and j. The Haversine formula defines the distance between population i and population j as

$$d_{ij} = 2R \arcsin\left(\sqrt{\sin^2\left(\frac{\omega_j - \omega_i}{2}\right) + \cos(\omega_i)\cos(\omega_j)\sin^2\left(\frac{\xi_j - \xi_i}{2}\right)}\right) \quad (2)$$

where R is the equatorial radius of the Earth ($R = 6378.145$ km), ω_k and ξ_k are the latitude and longitude, respectively, of population k. To calculate the Haversine distance, we obtained the latitudes and longitudes of each population from shape files obtained from the U.S. Census [3]. The parameters a and b determine the impact of the donor and recipient population sizes, respectively, on the number of commuters, and the parameter c determines the impact of the distance between populations on the number of commuters. The parameters a, b, and c are estimated by fitting this equation to the commuter data previously described. We obtain parameter estimates for a, b and c via nonlinear least squares (using the R function, `nls`) to minimize the objective functional

$$\sum_{\substack{i,j \\ i \neq j}} (m_{i,j} - \hat{m}_{i,j})^2 \quad (3)$$

where $m_{i,j}$ is the number of commuters from location i to location j as given by the U.S. Census commuter flow data and $\hat{m}_{i,j}$ is the model estimate of the number of commuters from location i to location j as given by Equation 1 for estimated values of a, b, and c. Note that because we are fitting the model to commuter data that considers movement among 161 locations, we are ultimately fitting the model to 161×160 total points.

The following values minimized the sum of squared errors: $a = 0.296$ (95% Confidence Interval: $0.2849, 0.3071$), $b = 0.437$ $(0.4262, 0.4480)$, $c = 0.749$ $(0.7300, 0.7671)$. S5 Fig shows the residual values as well as distributions of the residual values.

Characterization of *Ae. aegypti* seasonality

Because the CDC light traps utilized by Miami-Dade Mosquito Control in Miami-Dade county are not designed to target *Ae. aegypti*, counts in each of the traps for *Ae. aegypti* were generally low. *Ae. aegypti* were counted weekly in ten traps that remained in the same location during a four year period in which the data were collected (2010-2013). Of these ten traps, seasonal variation in the *Ae. aegypti* population was

apparent in eight. We used data from these eight traps to estimate the parameter τ_G in the following difference equation that describes the vector population dynamics each week (w) across a year:

$$\hat{N}_G(w+1) = \hat{N}_G(w) + \mu_G \hat{N}_G(w)\left(1 + \nu \cos\left(\frac{2\pi}{52}(w - \tau_G)\right)\right) - \mu_G \hat{N}_G(w) \tag{4}$$

Here, τ_G characterizes the time at which the vector population reaches its peak each season. We set $\mu_G = 1 - \exp(-7\hat{\mu}_G)$ to account for weekly dynamics, and fit this model to data for each of the eight traps to estimate values for $\hat{N}_G(1)$, ν, and τ_G by utilizing a nonlinear least squares approach (`lsqnonlin` in Matlab) to minimize the objective functional

$$\sum_{w=1}^{52} \left(N_G(w) - \hat{N}_G(w)\right)^2 \tag{5}$$

for each year. Here, $N_G(w)$ is the value given for the *Ae. aegypti* population in a trap on week w and $\hat{N}_G(w)$ is the model estimate for the *Ae. aegypti* population in a trap on week w. (Note that because the data represent weekly numbers of mosquito counts in each trap, we fit the model using an equation describing weekly population size; however, we scaled the parameter estimates for use in the full model, which is defined each day.) The average value of τ_G that we obtained across model fits for the eight traps was $\tau_G = 90.89$ days (range: 62.64-108.70 days, standard deviation: 14.26 days). We note that the estimates obtained for ν and $\hat{N}_G(1)$ are not important for the purpose of characterizing the *Ae. aegypti* population in the full epidemiological model because these values are specific to the absolute counts in each trap, which are not accurate representations of *Ae. aegypti* abundance. The population sizes in the full epidemiological model are much greater than those of each of the traps utilized in this part of the study. In S7 Fig, we show the fit of the vector population dynamic model to data for the eight traps utilized for estimating τ_G to characterize seasonality in the *Ae. aegypti* population (top 8 panels of S7 Fig) as well as the two traps that were not utilized in estimating τ_G (bottom 2 panels of S7 Fig). We note that trap SW2 was not utilized because of the four years of data provided, there were not enough data points in the third year to indicate any seasonality (bottom left panel of S7 Fig). Trap VK1 was not included because the data across the four year period was too sparse (only 9 total data points across 4 years, bottom right panel of S7 Fig). We note that we also combined all trap data to estimate τ_G and obtained the value of $\tau_G = 89.18$ days which did not differ significantly from the value obtained from the mean of the eight traps.

Parameter sensitivities

We explored the impacts of the movement scaling parameter θ on the probability of autochthonous transmission and the number of cases that occurred within 100 days of introduction. We allowed θ to vary uniformly (on a $\log_{10}$ scale) between 10^{-8} and 10^{-4}. As the value of θ increased, the probability of autochthonous transmission did not change dramatically until $\theta > 10^{-5}$ (S13 Fig A). The median of the cumulative distribution of the probability of autochthonous transmission then increased from about 0.6 at $\theta = 10^{-5}$ to about 0.9 when $\theta = 10^{-4}$. Changes in θ had a greater impact on the median number of cases that followed introduction (S13 Fig B). When $\theta = 10^{-6}$, the median value of the cumulative distribution for the median number of cases was between 100 and 1000 cases, but when θ increased beyond 10^{-6}, the median of the cumulative distribution for the number of cases following introduction rose by 3 orders of magnitude. For the highest values of θ we considered here, almost the entire population was becoming infected in more than 50% of simulations conducted because large values of θ lead to a loss of spatial structure such that the entire region is effectively one population.

Non-identifiability of Parameters

In the main text, we obtained plausible parameter sets from 5000 combinations of values for the average vector-host ratio, β, and θ. We found that only 154 of the 5000 sets were plausible given the case data and our range of assumed reporting rates. To further explore this, we conducted further simulations in a single homogeneously mixed population representative of the combination of two urban centers of Martin County where the 2013 dengue outbreak occurred [4]. The population size for this part of the study was 38000. We generated 3000 parameter combinations of β and the average vector-host ratio and ran 100 simulations for each parameter combination. Parameter values were chosen independently and randomly from uniform distributions: average vector-host ratio $\sim$ uniform$(0, 5)$, $\beta \sim$ uniform$(0, 1.5)$. S9 Fig shows the combinations of parameter values utilized in this study.

We found that in the 42 (1.4%) plausible parameter sets (i.e. parameter sets that led to a maximum number of cases from 100 simulations between 170-340), values for the two parameters were inversely related (black dots, S9 Fig). As the value of β increased, the average vector-host ratio decreased, with the latter decreasing rapidly for values of $\beta < 0.5$. This result indicates that identification of these two parameters is difficult with only the case data from the FDOH. These parameters are closely related, and estimation of β from the case data is difficult without an adequate understanding of the abundance of *Ae. aegypti* throughout the region that would provide a better estimate of the average vector-host ratio.

Calculation of Type Reproductive Number

The deterministic analog to the model presented in the main text is given by the following equations:

$$S_H(i,t+1) = S_H(i,t)(1-\lambda_H(i,t))$$

$$E_H(i,t+1) = E_H(i,t)(1-\sigma_H) + \lambda_H(i,t)S_H(i,t)$$

$$I_H(i,t+1) = I_H(i,t)(1-\gamma_H) + \sigma_H E_H(i,t)$$

$$R_H(i,t+1) = R_H(i,t) + \gamma_H I_H(i,t)$$

$$S_G(i,t+1) = S_G(i,t)(1-\mu_G)(1-\lambda_G(i,t)) + \psi_G(t)N_G(i,t)$$

$$E_G(i,t+1) = E_G(i,t)(1-\mu_G)(1-\sigma_G) + (1-\mu_G)\lambda_G(i,t)S_G(i,t)$$

$$I_G(i,t+1) = I_G(i,t)(1-\mu_G) + \sigma_G(1-\mu_G)E_G(i,t)$$

To calculate the expression for $R_0^T(i,t)$, we implement the next generation method for discrete time models [5,6]. With this method, R_0^T is the square of the spectral radius of the next generation matrix K, where

$$K = F(\mathbb{I}-V)^{-1} \tag{6}$$

Here, F describes new infections and V describes transitions between states.

$$F = \begin{pmatrix} 0 & 0 & 0 & \beta \\ 0 & 0 & 0 & 0 \\ 0 & \beta\frac{N_G(i,t)}{N_H(i)} & 0 & 0 \\ 0 & 0 & 0 & 0 \end{pmatrix}$$

$$V = \begin{pmatrix} (1-\sigma_H) & 0 & 0 & 0 \\ \sigma_H & (1-\gamma_H) & 0 & 0 \\ 0 & 0 & (1-\sigma_G)(1-\mu_G) & 0 \\ 0 & 0 & \sigma_G(1-\mu_G) & (1-\mu_G) \end{pmatrix}$$

References

[1] U. S. Census Bureau. 2010 Census Population and Housing Tables (CPH-Ts); 2010. Available: http://www.census.gov/population/www/cen2010/cph-t/cph-t-9.html.

[2] U. S. Census Bureau. OnTheMap; 2010. Available: http://onthemap.ces.census.gov/.

[3] U. S. Census Bureau. Tiger/Line Shapefiles and Tiger/Line Files; 2010. Available: https://www.census.gov/geo/maps-data/data/tiger-line.html

[4] Christofferson RC, Mores CN, Wearing HJ. Characterizing the likelihood of dengue emergence and detection in naive populations. Parasites & vectors. 2014 Jun;7(1):282.

[5] Heesterbeek JAP, Roberts MG. The type-reproduction number T in models for infectious disease control. Mathematical Biosciences. 2007 Mar;206(1):3-10.

[6] Allen LJS, van den Driessche P. The basic reproduction number in some discrete-time epidemic models. Journal of Difference Equations and Applications. 2008 14(10-11):1127-1147
