# Supplementary figures and images for: Modeling Mosquito-Borne Disease Spread in U.S. Urbanized Areas: The Case of Dengue in Miami
Source: PLoS One. 2016 Aug 17;11(8):e0161365. doi: 10.1371/journal.pone.0161365 (PMC4988691; doi:10.1371/journal.pone.0161365)

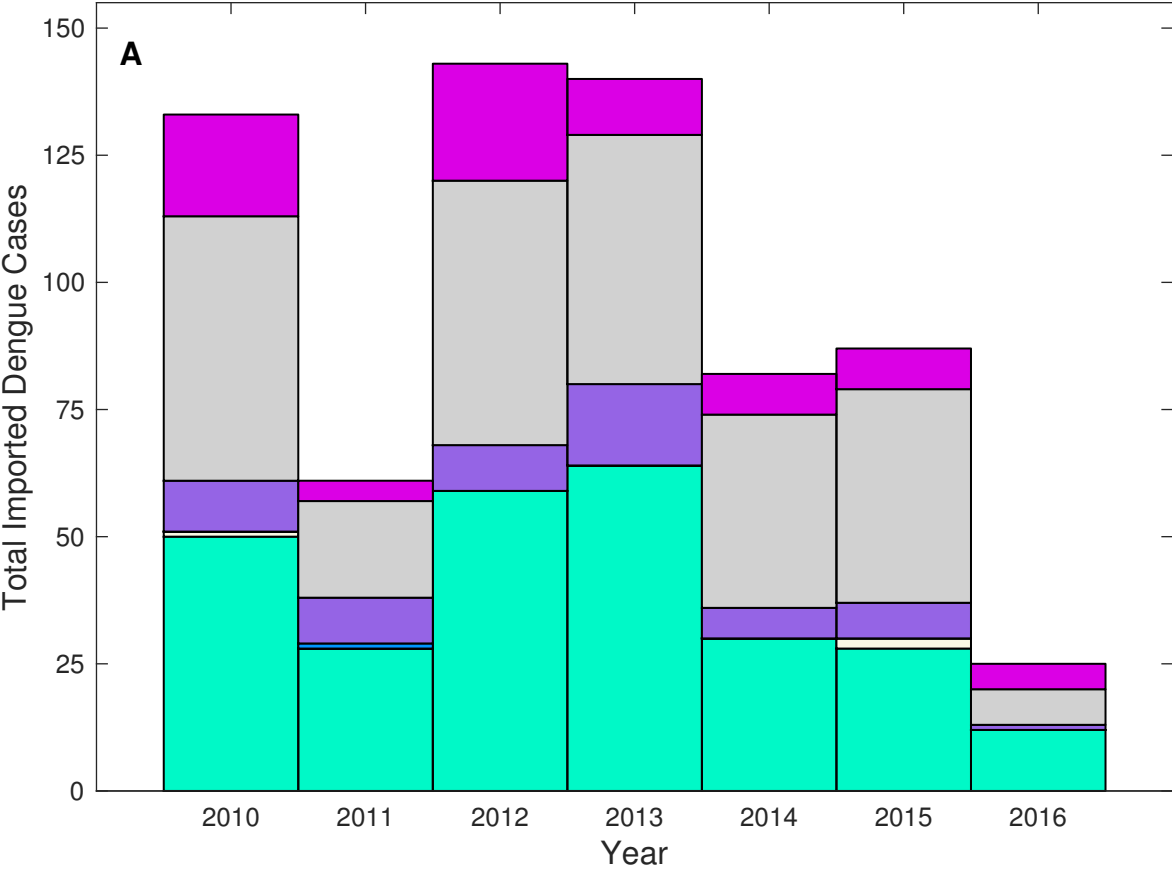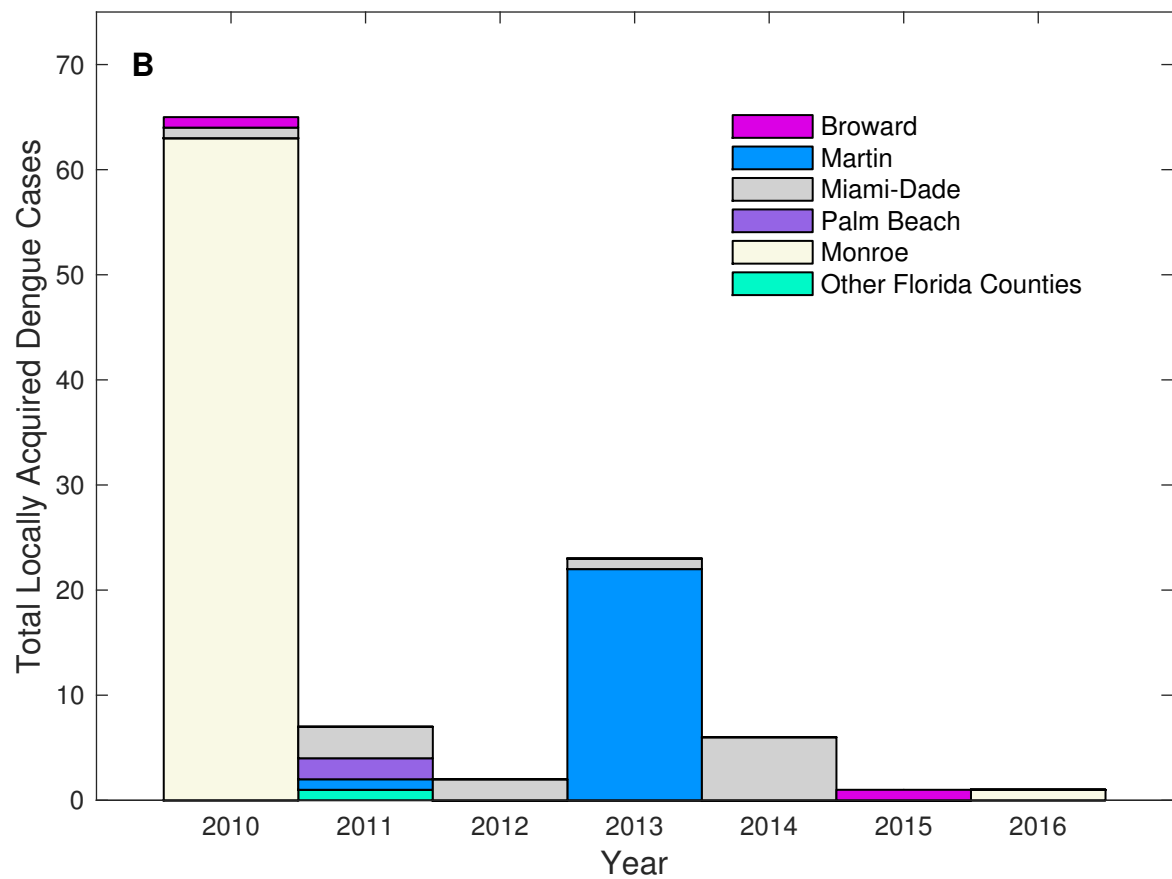

Supplement: S1 Fig — Total cases of dengue imported (A) and locally acquired (B) in the Miami UA and Florida. Broward, Miami-Dade, and Palm Beach counties comprise the majority of the Miami UA. Martin County, part of which is considered to be within the Miami UA, is located just north of Palm Beach county. Monroe county includes the Florida Keys and is located west and southwest of the Miami UA. Data presented here were obtained from [37]. Note that 2016 numbers are as of June 15, 2016. (PDF) [file pone.0161365.s002.pdf]

**Miami UA**

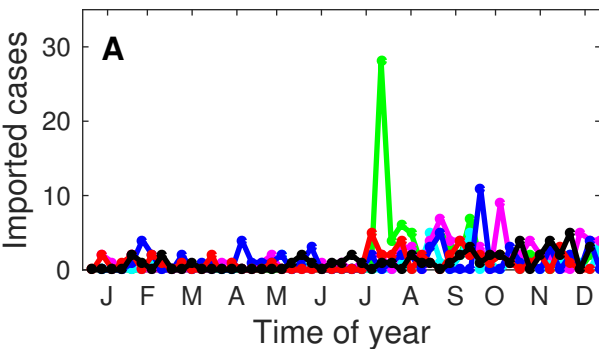

**Florida - Other**

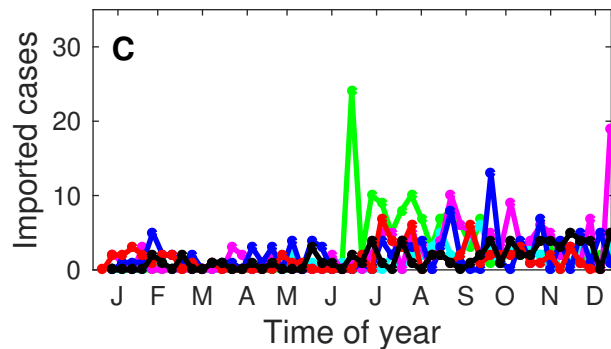

**Miami UA**

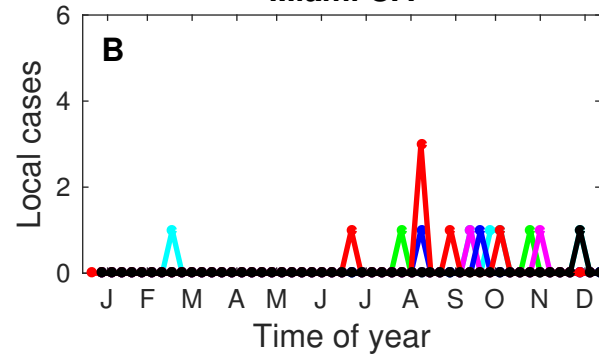

**Florida - Other**

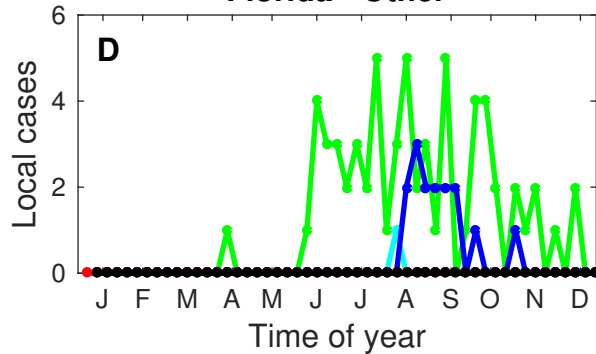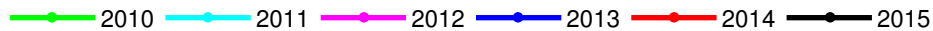

Supplement: S2 Fig — Time series of the number of imported (A,C) and locally acquired (B,D) cases of dengue in the Miami UA (Broward, Miami-Dade, and Palm Beach counties, A,B) and the remainder of Florida (C,D). Data presented in these figures were obtained from [37]. Each curve in the figure represents a different year (2010–2015). Note that the time associated with cases is the time at which Florida Department of Health reported the cases. (PDF) [file pone.0161365.s003.pdf]

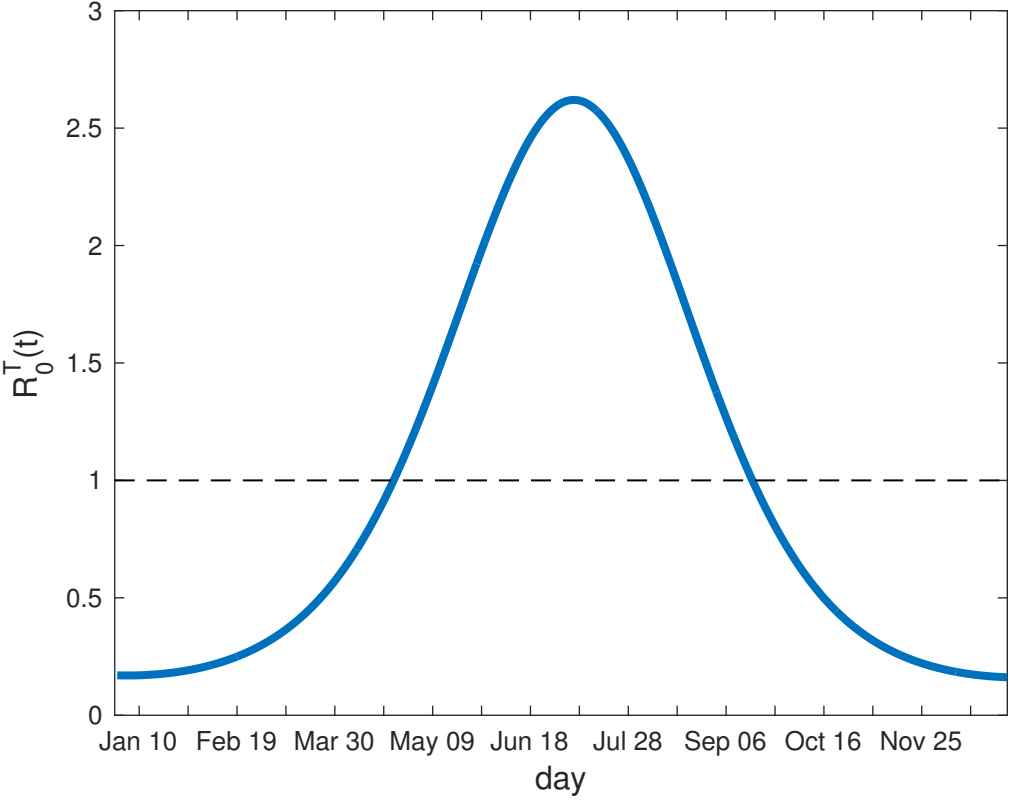

Supplement: S3 Fig — R0T(t) (not taking into account the influence of human movement) at different times of the year when the average vector-host ratio is 1. Note that this calculation assumes that the vector-host ratio remains the same from the day of introduction forward and is thus an approximation of the value of R0 on the day in which an imported case is introduced. The dashed black line indicates the epidemiological threshold value of R0T(t)=1. This R0T(t) value is calculated with the parameters listed in Table 1 of the main text. (PDF) [file pone.0161365.s004.pdf]

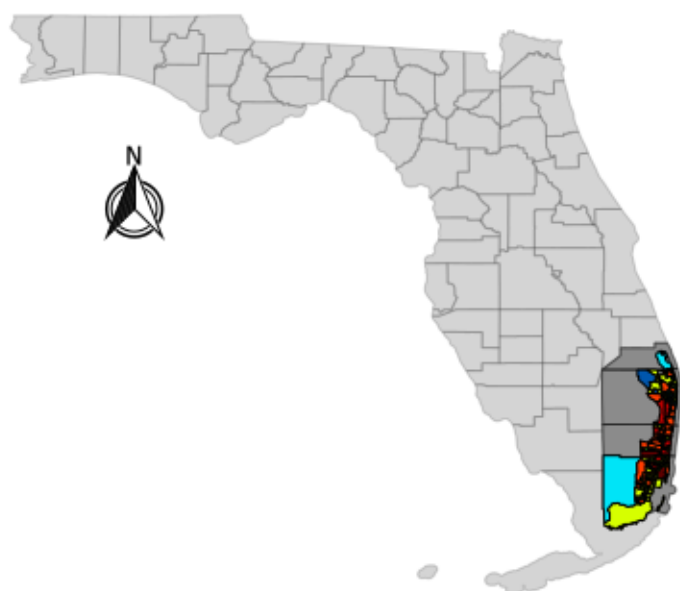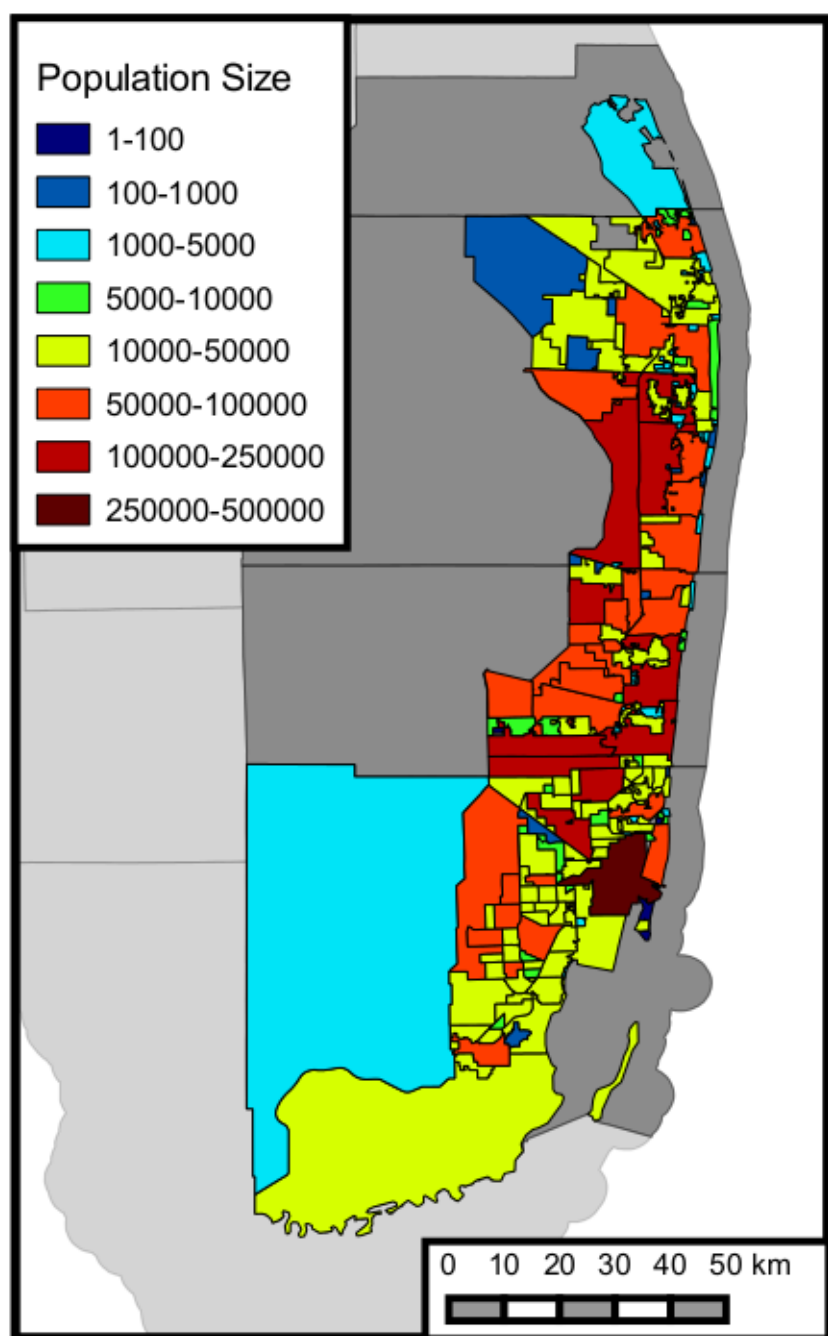

Supplement: S4 Fig — The counties in the Miami UA are, from top to bottom, Martin, Palm Beach, Broward, and Miami-Dade. The map included in this figure was obtained from U.S. Census Bureau TIGER/LINE®Shapefiles [71]. (PDF) [file pone.0161365.s005.pdf]

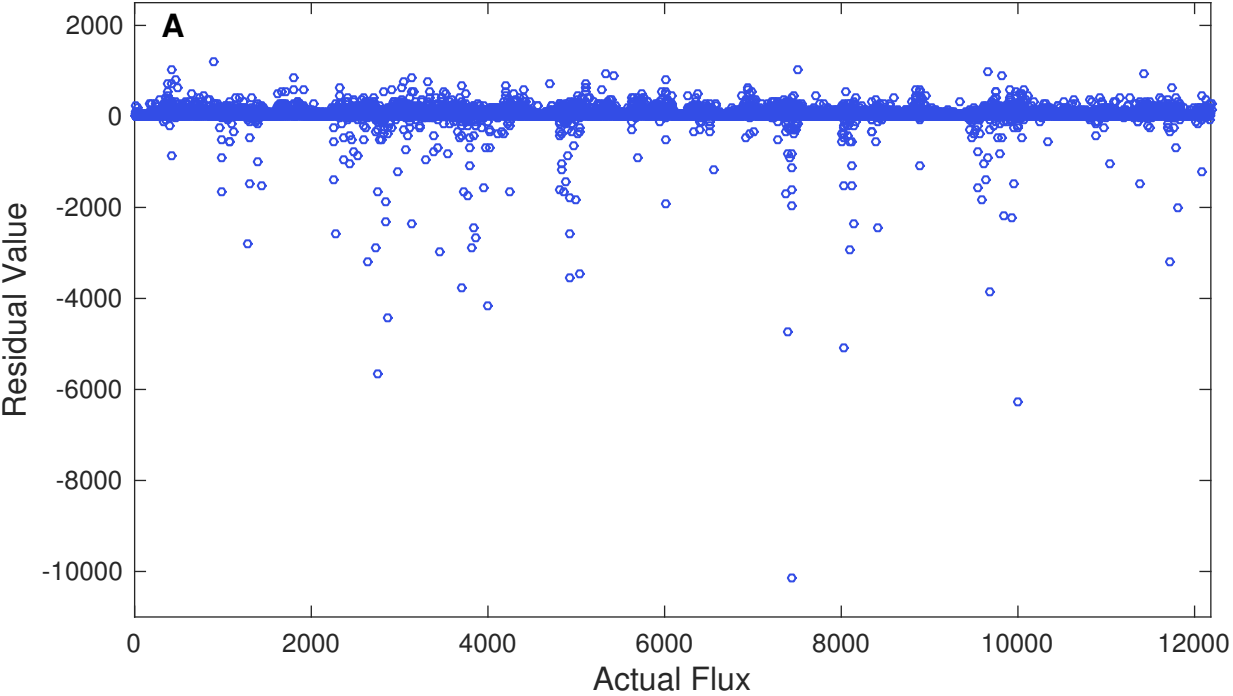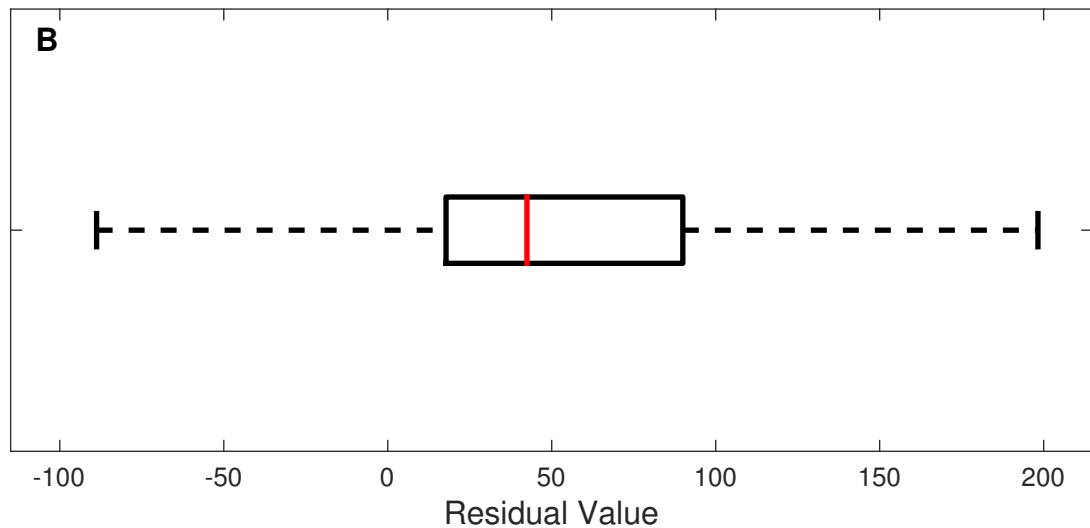

Supplement: S5 Fig — In the boxplot, the red line represents the median value and the box represents the interquartile range. (PDF) [file pone.0161365.s006.pdf]

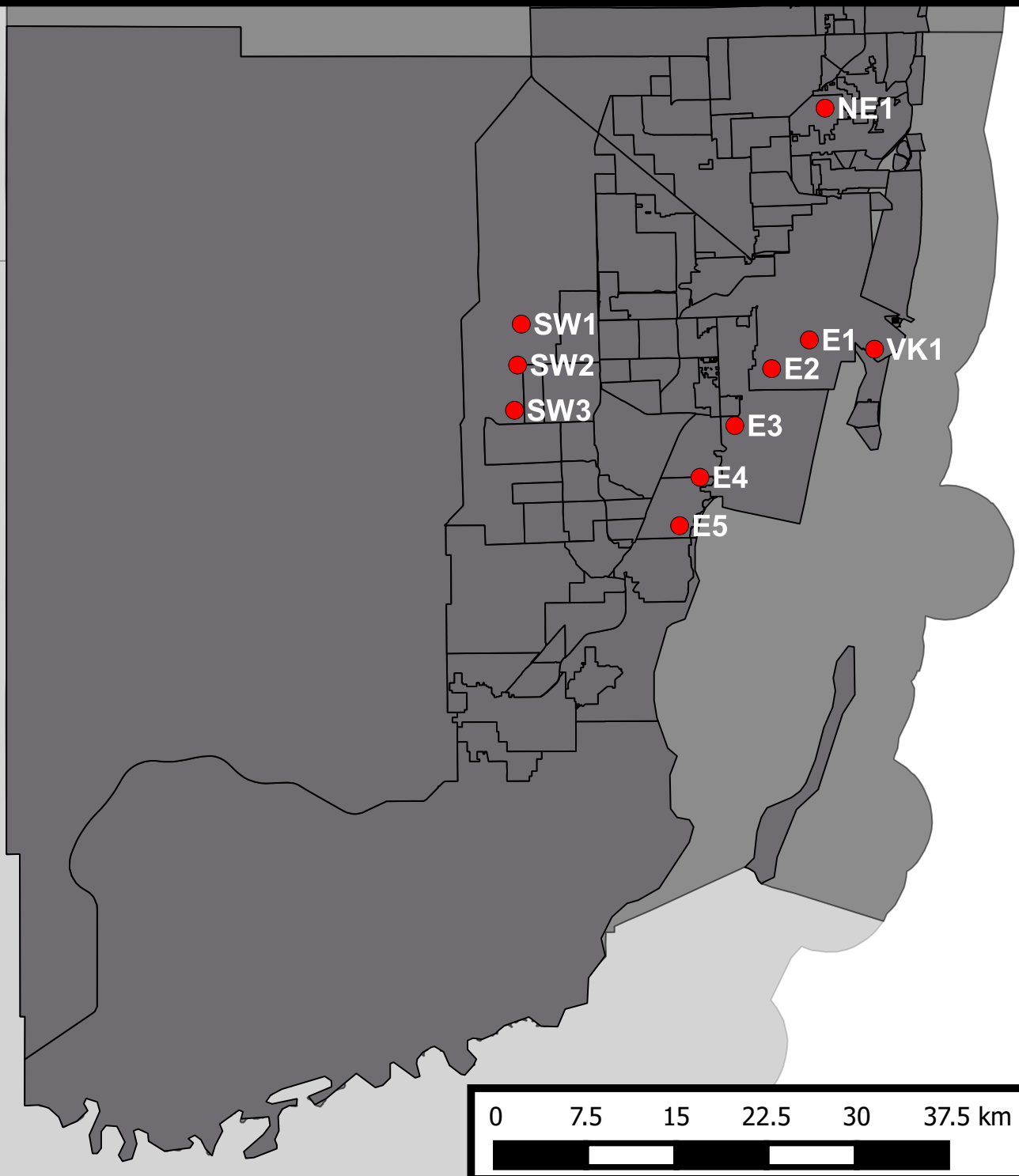

Supplement: S6 Fig — The map included in this figure was obtained from U.S. Census Bureau TIGER/LINE®Shapefiles [71]. (PDF) [file pone.0161365.s007.pdf]

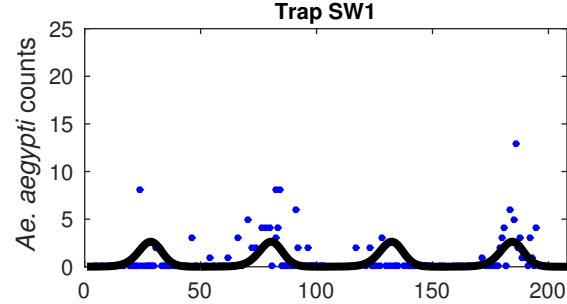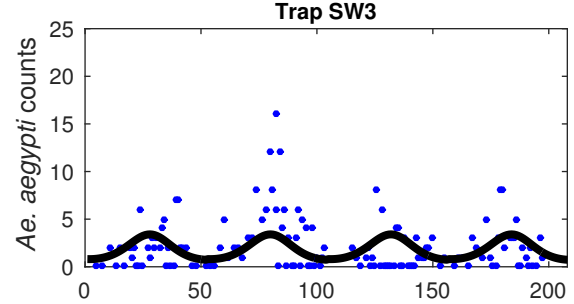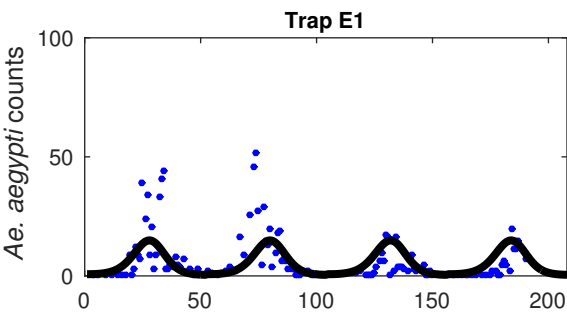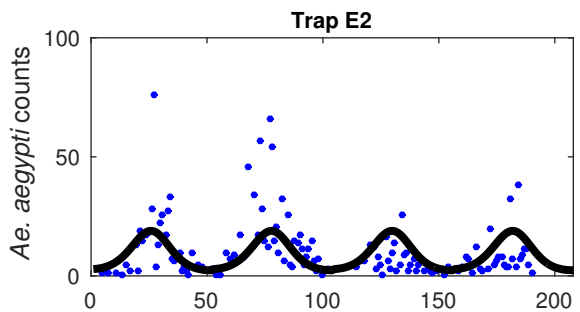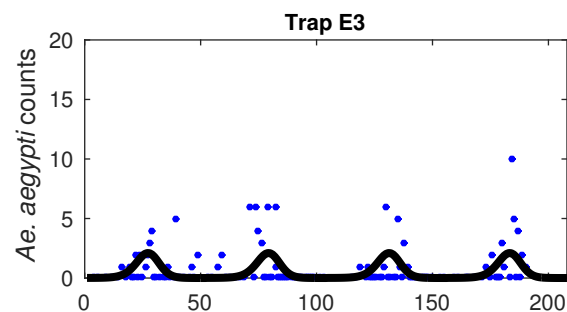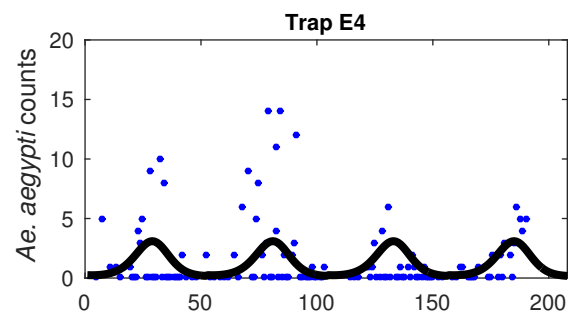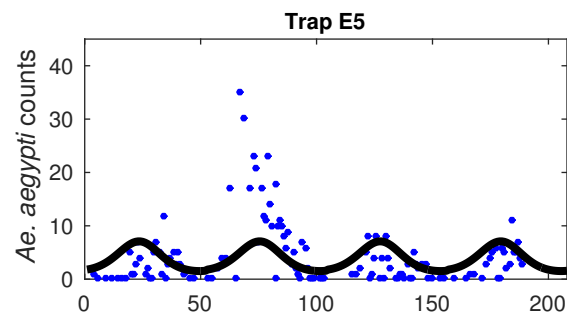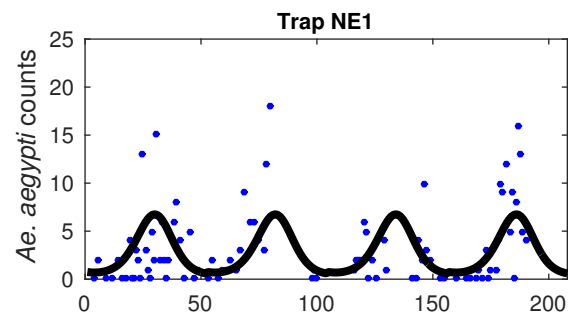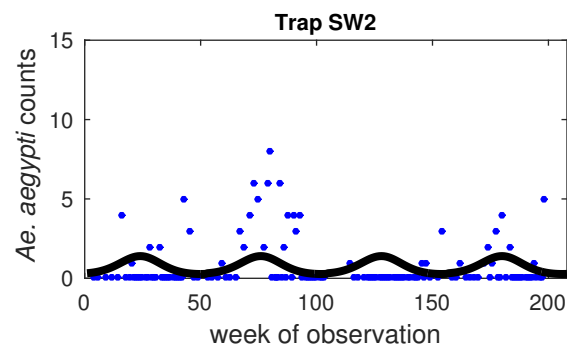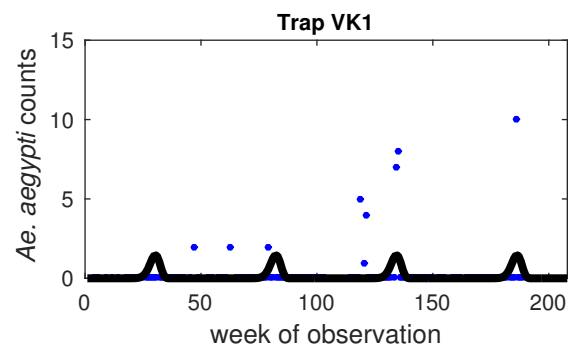

Supplement: S7 Fig — Blue dots are weekly counts and the solid black line is Eq S4 fit to each trap using the data presented in each figure. (PDF) [file pone.0161365.s008.pdf]

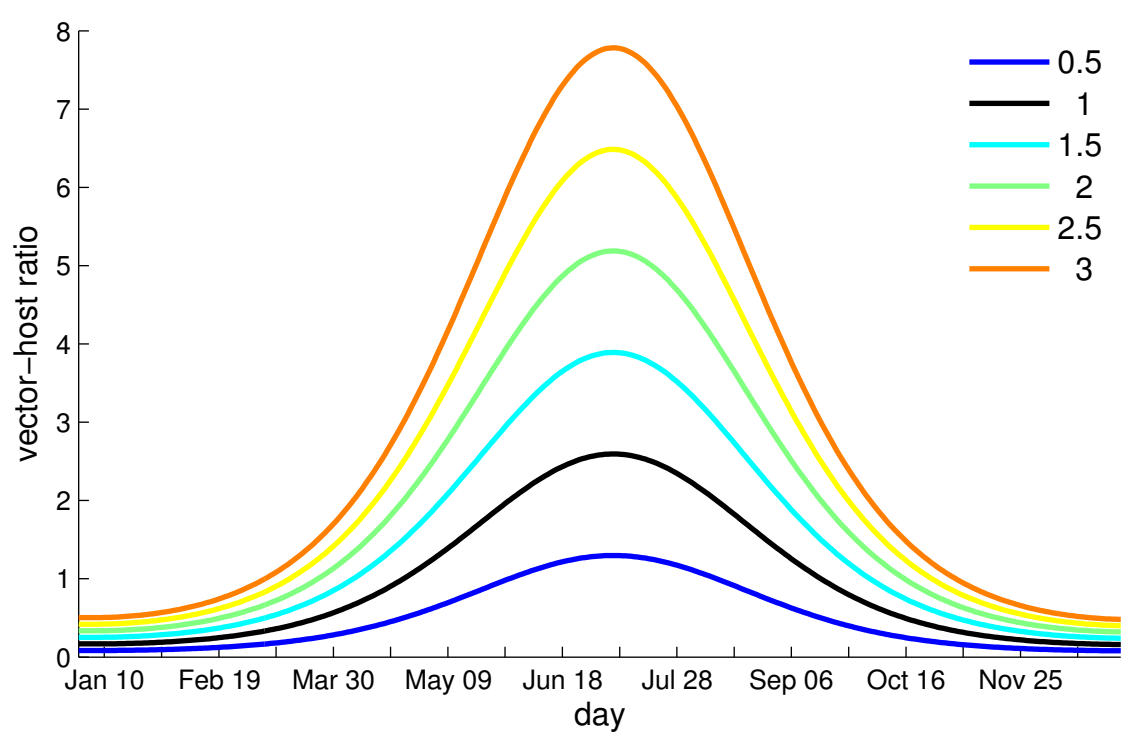

Supplement: S8 Fig — The curves represent the vector-host ratio as it changes throughout the year with fluctuations in the vector population. Each curve represents a deterministic simulation with a different value for the average vector-host ratio. In the main text, the average vector-host ratio is 1, which is indicated by the black curve in this figure. (PDF) [file pone.0161365.s009.pdf]

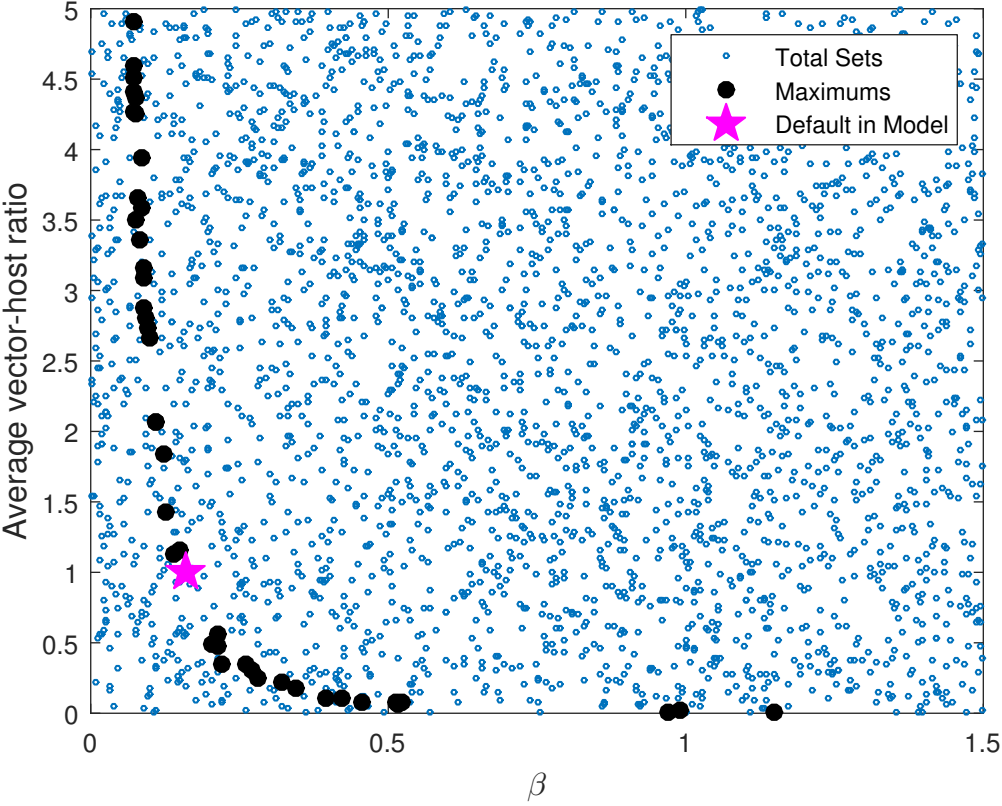

Supplement: S9 Fig — Scatter plot of 3000 combinations of values of β and the average vector-host ratio utilized in simulations of dengue introduction in a single homogeneous population of size 38000. Light blue circles represent all combinations of the two parameters. Dark black circles represent the values of the two parameters that were deemed plausible (i.e. led to a maximum number of cases from 100 simulations between 170–340). The pink star represents the combination of the two parameters utilized as the default values in this study. For more information, see S1 Text. (PDF) [file pone.0161365.s010.pdf]

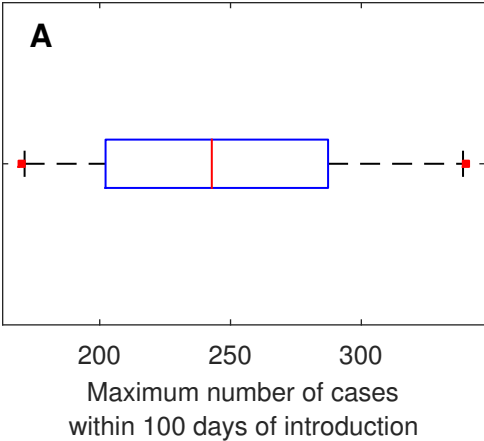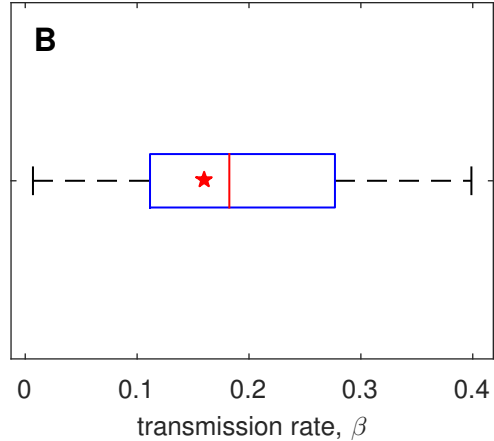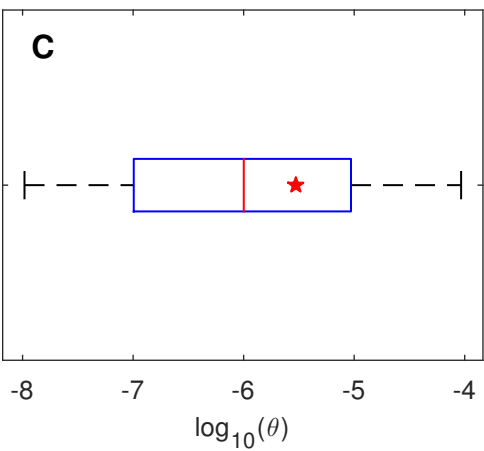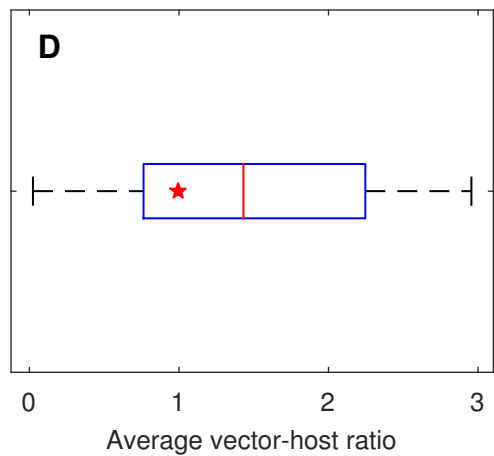

Supplement: S10 Fig — (A) The maximum number of cases from 100 simulations of each parameter set. The red squares denote the upper (340) and lower (170) bounds on the maximum number of infections required for a parameter set to be deemed plausible. (B) β. (C) θ (log scale), and (D) The average vector-host ratio. For panels (B-D), the red star represents the default value chosen for that parameter from one plausible parameter set. The red line represents the median, and the box encases the Interquartile range (IQR). The whiskers indicate 1.5×IQR. (PDF) [file pone.0161365.s011.pdf]

**A**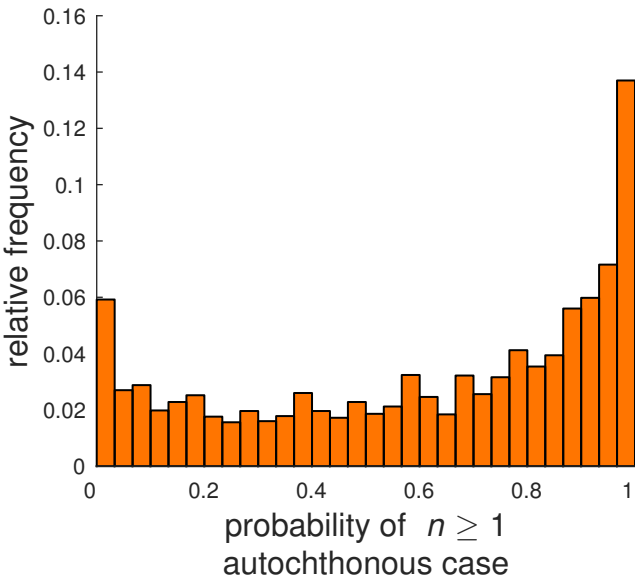**B**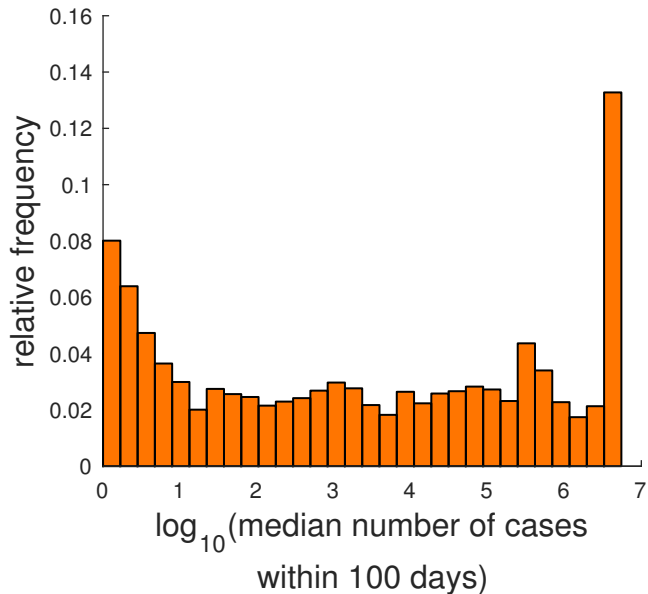

Supplement: S11 Fig — (A) The relative frequency of the probability of at least one autochthonous case. (B) The relative frequency of the median number of cases that occurred within 100 days of introduction (on a log10 scale). For these simulations, the average vector-host ratio was chosen from a uniform distribution with minimum 0 and maximum 3, β ∼ uniform(0, 0.4), and log10(θ) ∼ uniform(-8, -4). All other parameter values are as given in Table 1 of the main text. (PDF) [file pone.0161365.s012.pdf]

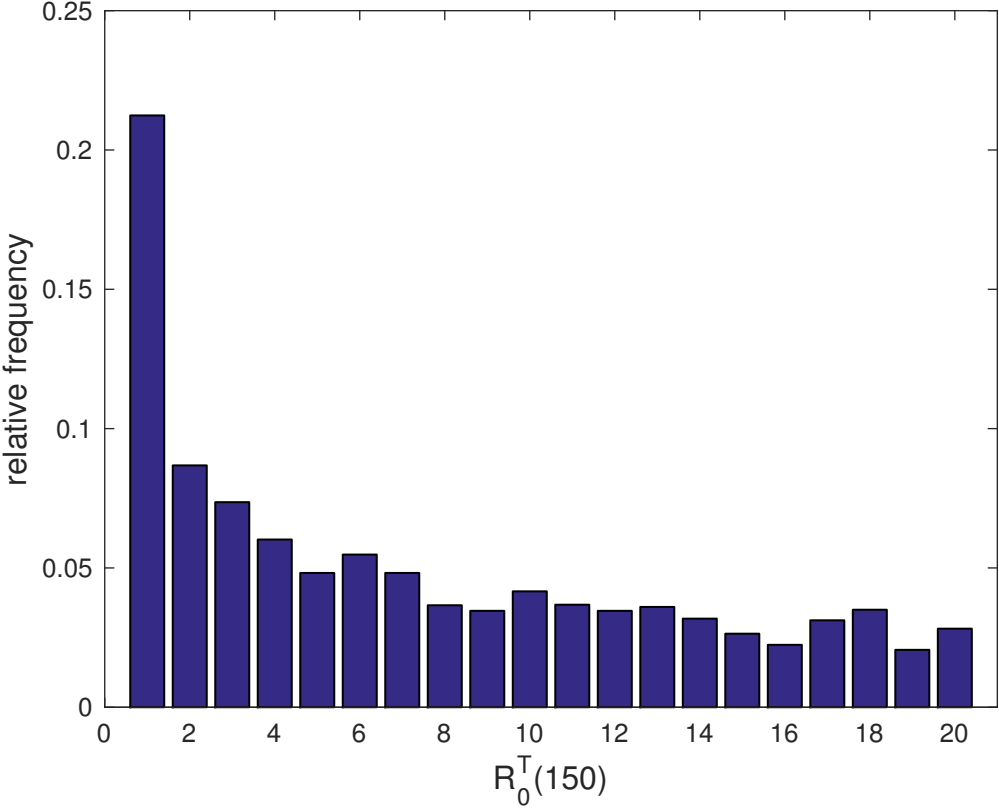

Supplement: S12 Fig — The relative frequency of R0T(t) on May 30. For these simulations, the average vector-host ratio was chosen from a uniform distribution with minimum 0 and maximum 3, β ∼ uniform(0, 0.4), and log10(θ) ∼ uniform(-8, -4). All other parameter values are as given in Table 1 of the main text. (PDF) [file pone.0161365.s013.pdf]

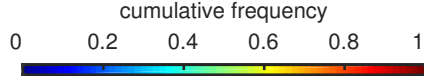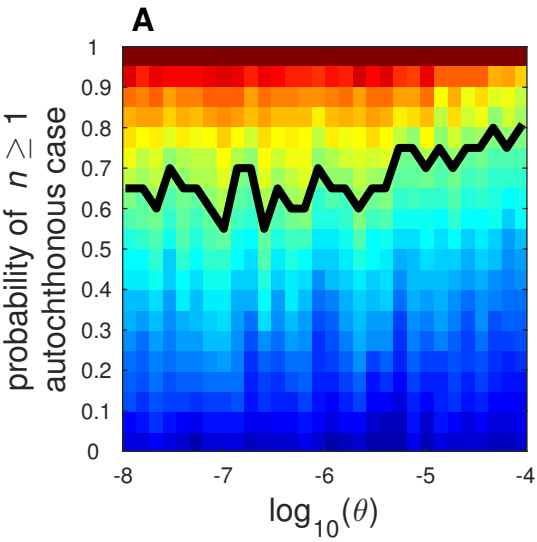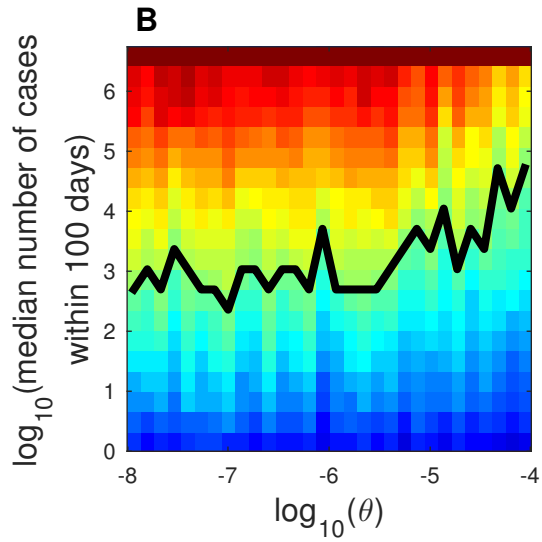

Supplement: S13 Fig — Heat maps depict how the probability of autochthonous transmission (A) changes with changes in θ and how the median number of cases that occurred within 100 days of introduction (B) changes with changes in θ. Note that the axis for the median number of cases that occur within 100 days is on a log10 scale. In each heat map, the parameters on the horizontal axis are divided into 30 evenly spaced groups and the values on the vertical axis are divided into 20 evenly spaced groups. The colored rectangles represent the cumulative frequency of simulations conducted with parameter values in the group on the horizontal axis that led to values of the metric in the group on the vertical axis. The solid black curve represents the median of the cumulative distribution for the metric presented on the vertical axis for each group of values on the horizontal axis. These figures were generated from 5000 total simulation sets. Each simulation set is a unique parameter combination that is run 100 times. For these simulations, the average vector-host ratio ∼ uniform(0, 3), β ∼ uniform(0, 4), and log10(θ) ∼ uniform(−8, −4). All other parameter values are as given in Table 1 of the main text. (PDF) [file pone.0161365.s014.pdf]

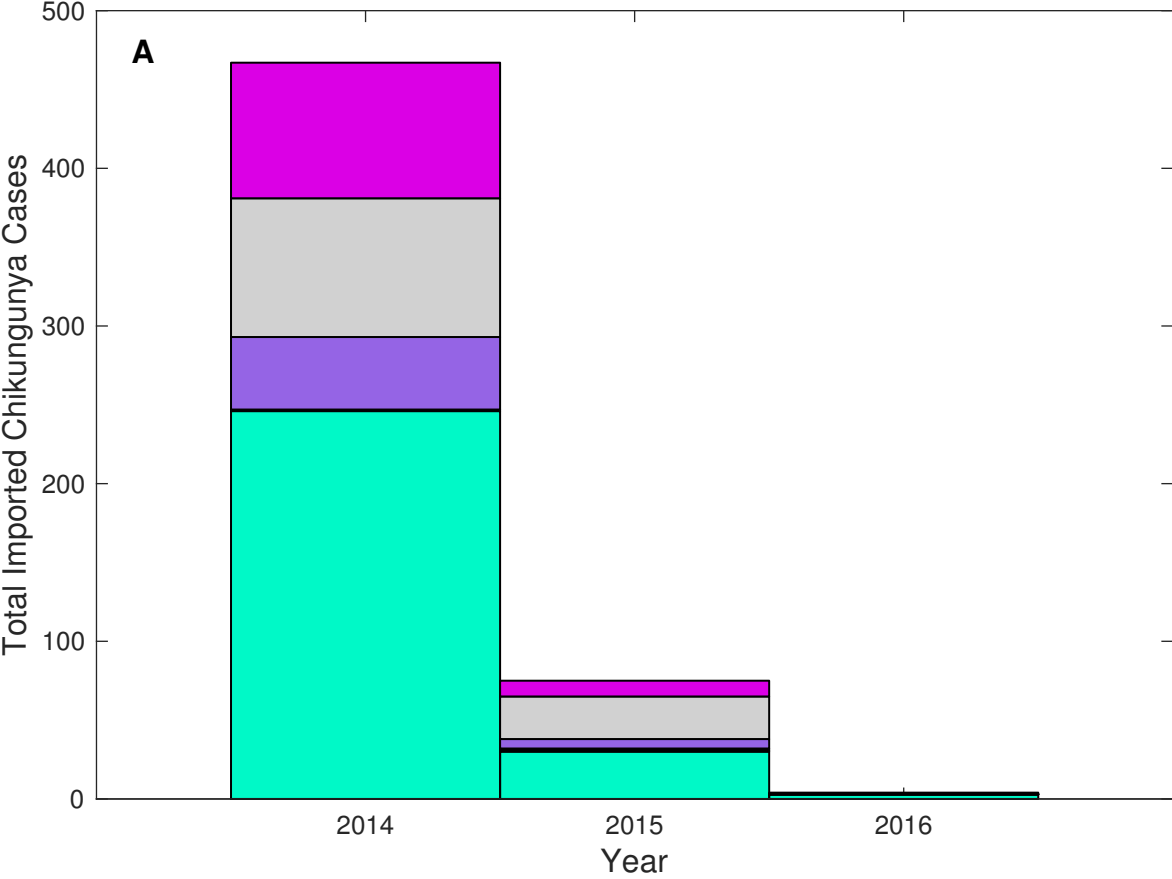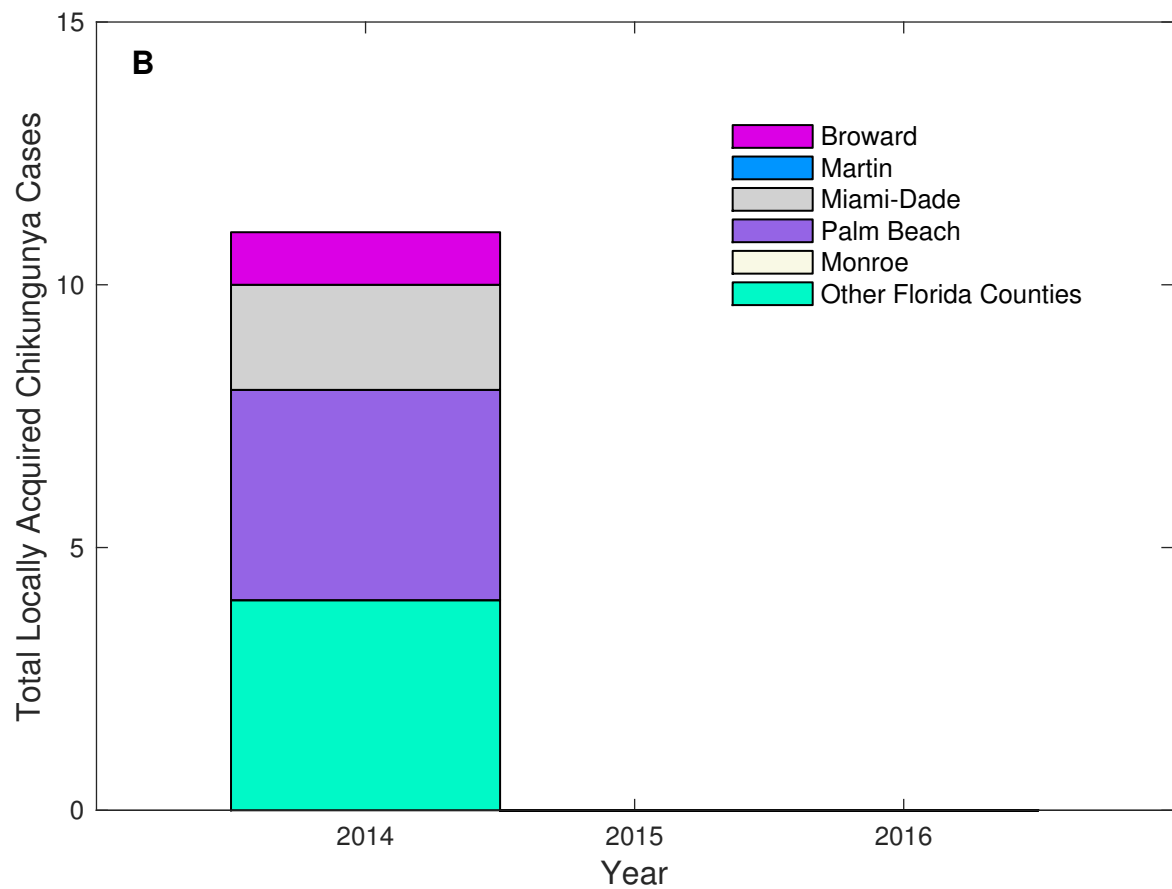

Supplement: S14 Fig — Imported (A) and locally acquired (B) chikungunya cases in the Miami UA and Florida. Broward, Miami-Dade, and Palm Beach counties are part of the Miami UA. Martin County, part of which is considered to be within the Miami UA, is just north of Palm Beach County. Monroe county includes the Florida Keys and is west and southwest of the Miami UA. Data presented in these figures were aggregated from [37]. Note that 2016 numbers are as of June 15, 2016. (PDF) [file pone.0161365.s015.pdf]

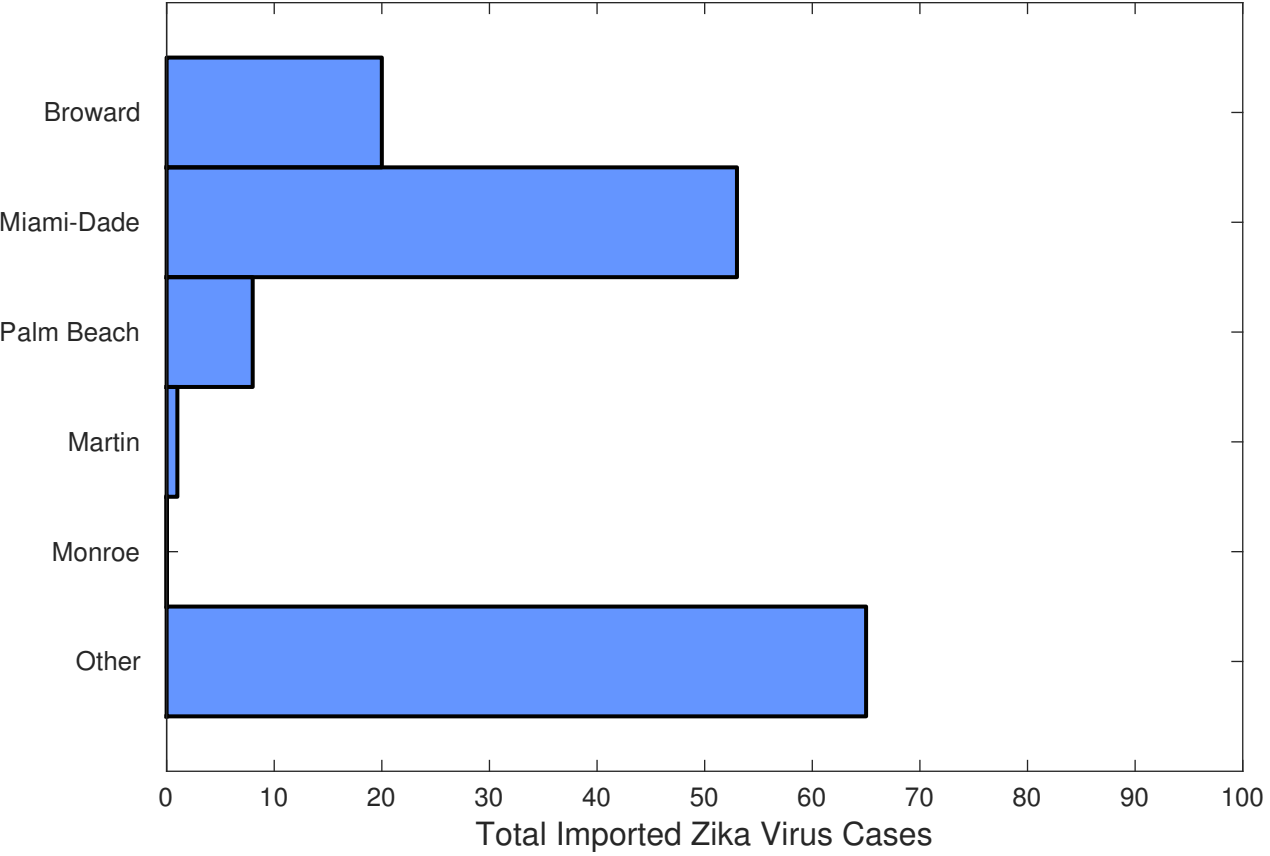

Supplement: S15 Fig — Imported Zika virus cases in the Miami UA and Florida. Broward, Miami-Dade, and Palm Beach counties are part of the Miami UA. Martin County, part of which is considered to be within the Miami UA, is just north of Palm Beach County. Monroe county includes the Florida Keys and is west and southwest of the Miami UA. Data presented in these figures were aggregated from [37]. Note that numbers are as of June 15, 2016. (PDF) [file pone.0161365.s016.pdf]
